# Parkinsonism mutations in *DNAJC6* cause lipid defects and neurodegeneration that are rescued by Synj1

**DOI:** 10.1038/s41531-023-00459-3

**Published:** 2023-02-04

**Authors:** Julie Jacquemyn, Sabine Kuenen, Jef Swerts, Benjamin Pavie, Vinoy Vijayan, Ayse Kilic, Dries Chabot, Yu-Chun Wang, Nils Schoovaerts, Nikky Corthout, Patrik Verstreken

**Affiliations:** 1 VIB-KU Leuven Center for Brain & Disease Research, 3000 Leuven, Belgium; 2 KU Leuven, Department of Neurosciences, Leuven Brain Institute, Mission Lucidity, 3000 Leuven, Belgium; 3 VIB-Bioimaging Core, 3000 Leuven, Belgium; 4 Present Address: Neuroscience and Mental Health Institute, University of Alberta, Department of Physiology, Department of Cell Biology, Group on Molecular and Cell Biology of Lipids, Edmonton, Alberta Canada; 5 Present Address: VIB Technology Watch, Technology Innovation Laboratory, VIB, Gent, Belgium

**Keywords:** Cell biology, Parkinson's disease

## Abstract

Recent evidence links dysfunctional lipid metabolism to the pathogenesis of Parkinson’s disease, but the mechanisms are not resolved. Here, we generated a new *Drosophila* knock-in model of *DNAJC6/Auxilin* and find that the pathogenic mutation causes synaptic dysfunction, neurological defects and neurodegeneration, as well as specific lipid metabolism alterations. In these mutants, membrane lipids containing long-chain polyunsaturated fatty acids, including phosphatidylinositol lipid species that are key for synaptic vesicle recycling and organelle function, are reduced. Overexpression of another protein mutated in Parkinson’s disease, Synaptojanin-1, known to bind and metabolize specific phosphoinositides, rescues the *DNAJC6/Auxilin* lipid alterations, the neuronal function defects and neurodegeneration. Our work reveals a functional relation between two proteins mutated in Parkinsonism and implicates deregulated phosphoinositide metabolism in the maintenance of neuronal integrity and neuronal survival.

## Introduction

Vesicle budding is a vital eukaryotic process controlling the shuttling of cargo and various types of lipids from the ER-Golgi network or the plasma membrane to other organelles^1,2^. Specifically in neurons, the synaptic delivery of proteins and lipids that are produced in the ER-Golgi complex, depends on vesicle trafficking. Neurons also extensively use Clathrin-mediated endocytosis (CME) at their nerve terminals, to recycle synaptic vesicles (SV) and to maintain chemical communication^3–6^. This is particularly relevant in Parkinsonism where mutations in the key CME-regulators Auxilin (*DNAJC6*, PARK19), Synaptojanin-1 (*SYNJ1*, PARK20) and EndophilinA-1 (*SH3GL2*) were identified to cause disease, and also other accessory regulatory proteins to CME like LRRK2, RME-8, GAK (Auxilin-II) are implicated^7–12^. However, how mutations in these proteins cause neuronal problems that are relevant to Parkinsonism, and whether there are functional relations and interactions between key “CME genes” mutated in Parkinsonism, remains poorly understood.

**Fig. 1:**
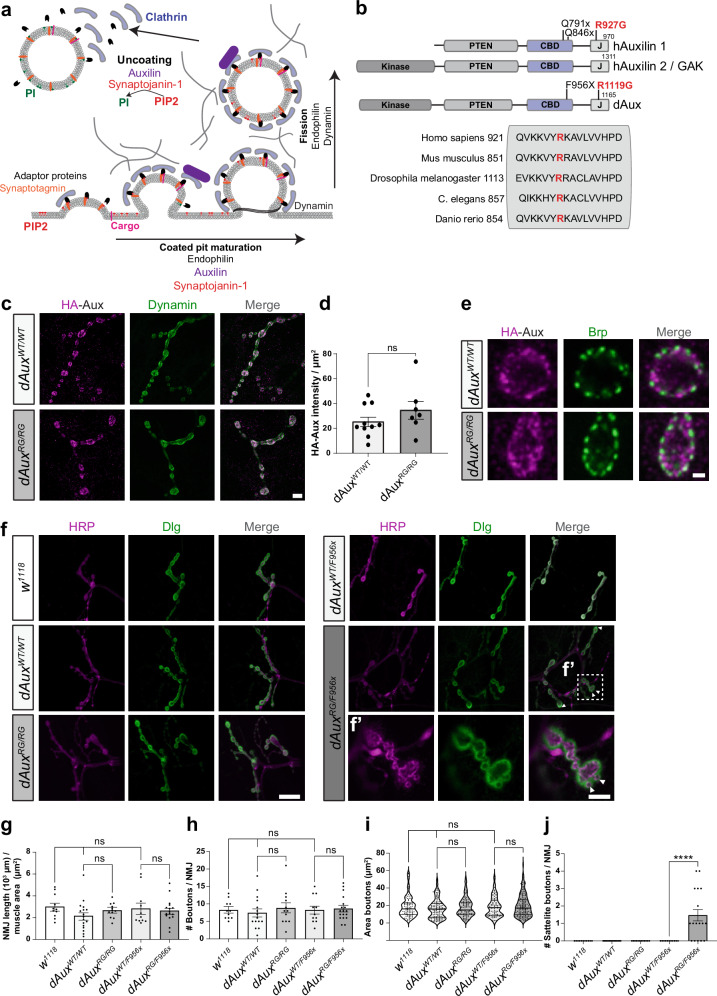
Auxilin protein level, localization and synaptic morphology are normal in pathogenic *dAux* mutant larvae. **a** Schematic of Clathrin-mediated-vesicle formation (here: endocytosis) (CME) indicating key proteins and lipids; conversion of phosphoinositides, phosphatidylinositol 4,5-bisphosphate [PI(4,5)P_2_] and phosphatidylinositol 3,4-bisphosphate [PI(3,4)P_2_] (PIP2). **b** Domain structure of mammalian and *Drosophila* Auxilin and pathogenic mutations mentioned in this work. The arginine in human Auxilin that is mutated in PD, at position 927 (red) is conserved across species and homologous to *Drosophila* R1119. **c**–**d** HA-tagged dAux^WT^ and dAux^RG^ proteins localize to the nerve terminals of the larval NMJs where they show overlap with Dynamin^37^. (**c**) Representative images of NMJs labelled with anti-HA (magenta, dAux) and anti-Dynamin (green) and (**d**) quantification of the mean HA-Aux intensity per μm^2^. n≥8 larvae (of 3 independent crosses). Bars show the mean ± SEM, points show individual values. Student’s *t*-test, ns: not significant. Scale bar: 2.5 µm. **e** Confocal images of L3 larval NMJs of *dAux*^*WT/WT*^ and *dAux*^*RG/RG*^ labelled with anti-Brp and anti-HA (magenta, dAux). Scale bar: 1 µm. **f**–**j** Confocal images of NMJs of *w*^*1118*^, *dAux*^*WT/WT*^, *dAux*^*RG/RG*^, *dAux*^*WT/F956x*^ and *dAux*^*RG/F956x*^ larvae labelled with anti-Hrp and anti-Dlg (**f**) and quantification (**g**–**j**). (**f’**) Enlargement of the indicated panel, white arrow: satellite boutons. Quantification of NMJ length per muscle area (**g**), bouton number per NMJ (**h**), size of individual boutons (**i**) and total number of satellite boutons per NMJ (**j**). *n* = 3 larvae (of 3 independent crosses). Dots represent the result of single NMJs (**g**–**j**) or area of single boutons (**i**). Bars show the group mean ± SEM, points show individual values. One-way ANOVA, with Dunnett’s multiple comparison test. ns: not significant, ***p < 0.001. Scale bars left 20 µm; right 5 µm.

Auxilin and Synj1 act in related steps of CME. During endocytosis, nascent vesicles can form because coat proteins like Clathrin and adaptors help to shape the synaptic plasma membrane. Auxilin is a co-chaperone to Hsc-70 and is involved in rearranging the Clathrin coat to remove it following vesicle formation (Fig. 1a)^13–16^. Synj1 is a phosphoinositide (PI) phosphatase that dephosphorylates vesicle membrane PIs causing the dissociation of proteins, like adaptors, that link the Clathrin-lattice to the membrane bilayer (Fig. 1a)^16–19^. Interestingly, Auxilin also contains a motif to bind mono-PIs in its PTEN domain (Fig. 1b). This suggests that Synj1-activity could promote Auxilin-recruitment to the membrane in the process of CME (Fig. 1a).

While these functional studies relied on severe loss-of-function or null mutants that cause lethality^19–24^, pathogenic mutations in *DNAJC6* and in *SYNJ1* cause only partial inactivation of protein function^25,26^. Interestingly, in patients, mutations in these genes cause early-onset, autosomal recessive Parkinsonism and result in a very similar spectrum of phenotypes that include Parkinson symptoms such as bradykinesia, resting tremor, rigidity and postural instability, as well as additional clinical problems including developmental delay and seizures that are not responsive to L-DOPA treatment^7–10,27–31^. However, the consequences of these mutations on synaptic integrity and neuronal survival during aging, and whether Auxilin and Synj1 can compensate for one another in the context of disease, remains enigmatic.

Creating new vesicles through CME involves topological membrane changes that depend on fine-tuned interactions between specific lipids and vesicle shaping- and budding-proteins. Recent observations recognize lipid disturbances in PD, including that there are changes in ceramide, sphingolipids, fatty acids, cholesterol and neutral lipids in postmortem brains of PD patients^32–34^. Whether these changes are primary defects or “end-stage” and thus confounded by the long disease process, is unclear. It is also not known what the functional consequences of such changes are on cellular and organellar function. However, it is conceivable that lipid alterations affect vesicle trafficking, thus connecting these to Parkinsonism.

Here, we find that a pathogenic Parkinsonism mutation, “RG”, knocked into the *Drosophila Auxilin/DNAJC6* locus causes in vivo lipid disturbances, neuronal dysfunction, and neurodegeneration. *Drosophila dAux*^*RG*^ mutants have lower levels of membrane lipids containing long-chain polyunsaturated fatty acids (LC-PUFA), including PI lipid species. Lipids containing LC-PUFA are suggested to modulate the localization and function of Synj^35,36^. Additionally, we show that Synj levels decline in animals with the “RG” mutation and that overexpression of Synj rescues the neuronal dysfunction induced in *dAux*^*RG*^, including behavioral defects and neurodegeneration. Furthermore, it also restores lipid defects. This work shows that the role of two Parkinsonism-linked genes is connected through neuronal lipid metabolism and suggests an important role for lipids in Parkinsonism pathogenesis.

## Results

### The pathogenic Auxilin mutant is a weak hypomorphic allele

Complete loss of Auxilin function causes early lethality, but partial loss-of-function causes neurological problems^9,10,20–22,24,27^. There are several pathogenic mutations that have been isolated in *DNAJC6*, including the R927G mutation in the J-domain that causes Parkinson motor problems^27^. The mutation is contained within the His-Pro-Asp (HPD) motif of the J-domain that is essential for Hsc70 recruitment (Fig. 1b). To model *DNAJC6/Aux*-induced disease, we created new *Drosophila* knock-in animals that express the pathogenic *Aux* mutation under endogenous promoter control. We first replaced the entire *dAux* gene by an AttP flanked *white*^*+*^ gene and then used ΦC31 to substitute the *white*^*+*^ marker by the entire *dAux*^*WT*^ gene or by the mutant *dAux*^*R1119G*^ gene (hereafter referred as *dAux*^*RG*^); both genes are 5′-HA-tagged (R1119G is homologous to the human pathogenic R927G mutation (Fig. 1b)).

To determine the impact of the R1119G mutation on synaptic function, we resorted to the third instar larval neuromuscular junction (NMJ) that is frequently used as a model synapse to study neuronal cell biology. We first examined the subcellular localization of dAux in *dAux*^*WT/WT*^ and *dAux*^*RG/RG*^ using anti-HA antibodies. dAux^WT^ and dAux^RG^ both localize to presynaptic nerve terminals (Fig. 1c, d), where they partially overlap with Dynamin, a known binding partner of Auxilin that acts in endocytosis (Fig. 1a)^37^. We also find dAux^WT^ and dAux^RG^ to be both enriched in the regions adjacent to active zones labelled by anti-Bruchpilot (Fig. 1e). These “peri-active zones” are sites of SV endocytosis^38^. This indicates that dAux localizes to synapses, similar to its mammalian counterpart^26,39^ and the R1119G mutation does not impair protein stability or localization at peri-active zones.

The appearance of supernumerary “satellite boutons” on Type 1b boutons is often seen in endocytic and membrane recycling mutants^19,40–44^. To test if *dAux* mutants have morphological defects at their NMJs, we labelled wild type controls (*w*^*1118*^), *dAux*^*WT/WT*^ and *dAux*^*RG/RG*^ with anti-HRP and anti-DLG that mark pre- and post-synaptic sites of NMJ boutons (Fig. 1f). Similar to *w*^*1118*^ and *dAux*^*WT/WT*^, satellite boutons do not appear in *dAux*^*RG/RG*^. Likewise, other morphological features, including the total number of boutons, bouton diameter, NMJ length or muscle surface area and length are not significantly different between these genotypes (Fig. 1f–j). Hence, at the level of our analysis, homozygous mutant *dAux*^*RG/RG*^ have normal synaptic morphology and do not suffer from a developmental delay.

To further investigate the nature of the R1119G mutation we performed a genetic analysis. We combined *dAux*^*RG*^ and *dAux*^*WT*^ with the severe hypomorphic *dAux*^*F956X*^ mutation^21^. *dAux*^*F956X*^ harbors a stop codon after the Clathrin binding domain, thus removing the J-domain (Fig. 1b). Synaptic morphology of *dAux*^*WT/F956X*^ and *dAux*^*RG/F956X*^ is very similar (Fig. 1f–i), except there are significantly more satellite boutons in *dAux*^*RG/F956X*^ (Fig. 1f, f’ white arrows and j). These data are consistent with *dAux*^*RG*^ being a weak hypomorphic (loss-of-function) allele whose phenotypes are revealed when combined with more severe loss-of-function alleles.

### Pathogenic Auxilin mutants harbor irregular-sized synaptic vesicles

To test if the Auxilin pathogenic mutant affects SV endocytosis, we quantified the uptake of FM 1–43 in live preparations; we dissected larvae to expose the neuromuscular junction, a model synapse to assay vesicle endocytosis. FM 1–43 is a lipophilic fluorescent dye that binds membranes and when added to this preparation, it is internalized in newly formed vesicle membranes by endocytosis upon stimulation (10 min, 90 mM KCl, 1.5 mM CaCl_2_). The amount of internalized fluorescence is a measure of endocytosis^45^. Under these conditions, no difference in dye uptake is observed between *w*^*1118*^ controls, *dAux*^*WT/WT*^, *dAux*^*RG/RG*^ *dAux*^*WT/F956X*^ and *dAux*^*RG/F956X*^ mutants (Supplementary Fig. 1a, b). To reveal the ultrastructure of the Auxilin mutant synapses we used transmission electron microscopy. We activated the vesicle cycle and thus endocytosis by first stimulating the neurons before processing and imaging (Supplementary Fig. 1c–g). We then implemented a machine learning approach to quantify the data in an unbiased manner (see Methods). Ultrastructural features such as pre-synaptic active zones and T-bars, and number of mitochondrial surface area (Supplementary Fig. 1c, d) and mitochondrial cristae structure are not different between the analyzed genotypes. Similarly, we do not find differences in SV number (<80 nm) or cisternae number (>80 nm) (Supplementary Fig. 1d–f) that are both parameters often found affected in endocytic mutants^46–51^. Next, we quantified the relative frequency of SV ( < 80 nm) diameter in bins of 5 nm. This reveals a more heterogeneous vesicle population, including a shift towards smaller SVs in *dAux*^*RG/RG*^ and in *dAux*^*RG/F956X*^ mutants, compared to *w*^*1118*^, *dAux*^*WT/WT*^, and *dAux*^*WT/F956X*^ controls (Supplementary Fig. 1d, g). While the FM 1–43 dye uptake experiment suggested that the total amount of membrane uptake during stimulation is not affected by the pathogenic Auxilin mutants, the irregularly sized vesicle population that forms during stimulation, is consistent with an impairment of efficient Clathrin coat re-arrangements during vesicle formation.

### The Auxilin R1119G mutation causes severe behavioral dysfunction

**Fig. 2:**
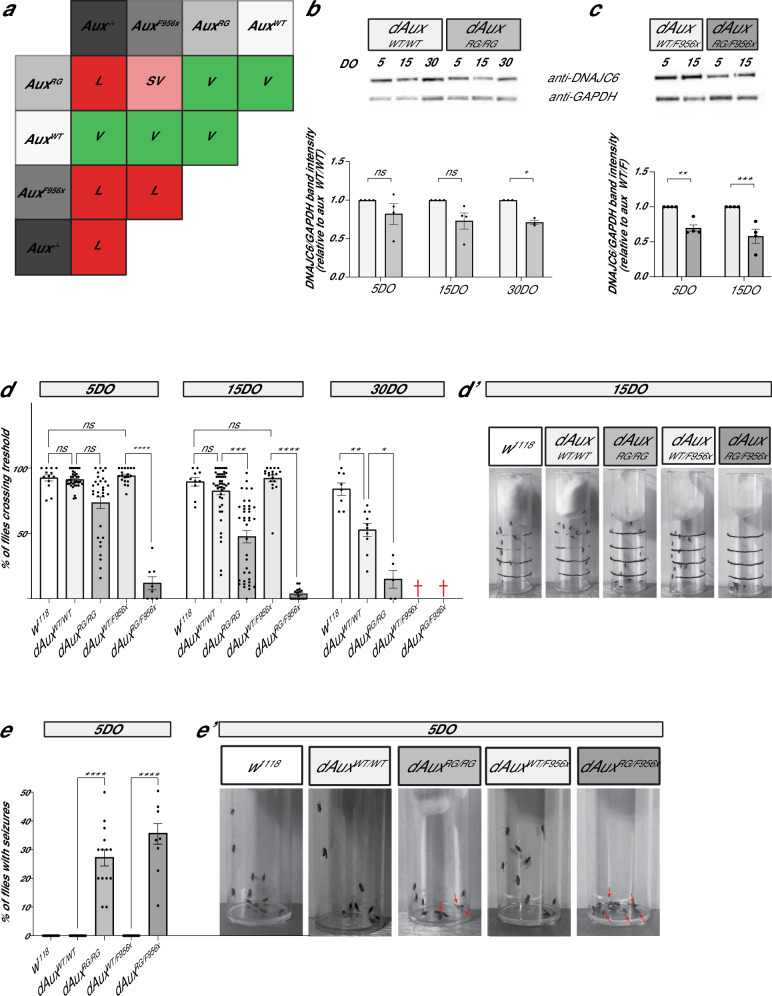
The pathogenic *Auxilin* mutants develop age-dependent neurological abnormalities. **a** Complementation table indicating viability/lethality of the indicated genotypes. L: lethal, SV: semi-viable and V: viable. **b**–**c**. Images of Western blots from brain lysates prepared from 5, 15 and 30DO flies of indicated genotypes labelled with anti-DNAJC6/Auxilin and anti-GAPDH (loading control) and quantification of Auxilin protein levels. Values are relative to GAPDH and are expressed as a fraction of protein levels in *dAux*^*WT/WT*^ and *dAux*^*WT/F*^. Bars show the mean ± SEM, points show individual values and *n*≥3. Two-way ANOVA, with Bonferroni’s post hoc test. *p < 0.05, **p *<* 0.01 and ***p < 0.001. **d**–**d**’. Quantification of the negative geotaxis assay of 5DO, 15DO or 30DO flies of the indicated genotypes (**d**) and representative images of 15DO flies after 30 s (**d’**). Graphs represent the fraction of flies crossing the 4 cm mark. Note that most *dAux*^*WT/F956X*^ and all *dAux*^*RG/F956X*^ flies die within 25-days-post-eclosion (red crosses). Bars show mean ± SEM. Points represent the mean of three trials each. One-way ANOVA, Dunnett’s multiple comparison test. ns: not significant, *p < 0.05, **p < 0.01, ***p < 0.001 and ****p < 0.0001. † dead at this age. **e**–**e**’. Quantification of seizures following vortexing in 5DO flies of indicated genotypes (**e**) and representative images of flies after vortex-stimulation (**e’**). Graphs represent the fraction of flies that were unable to stand 10 s after stimulation. Bars show mean ± SEM. Points represent single trials with 10 flies each. Red arrows: flies that are not able to stand. One-way ANOVA, Dunnett’s multiple comparison test. ns: not significant, ****p < 0.0001.

The larval NMJ is a very robust synapse that may not reveal the more subtle problems that may arise during aging in central nervous system neurons. We therefore turned to adult flies and performed a genetic complementation analysis using *dAux*^*WT*^, *dAux*^*RG*^, *dAux*^*F956X*^ and *dAux*^*-*^ null mutant flies. While *dAux*^*–/–*^, *dAux*^*F956x/–*^ and *dAux*^*RG/–*^ are lethal, a few *dAux*^*RG/F956X*^ mutant animals escape lethality, but all these flies die within 25-days-post-eclosion. Conversely, *dAux*^*WT/-*^, *dAux*^*WT/F956X*^, *dAux*^*WT/RG*^ *and dAux*^*RG/RG*^ flies are viable (Fig. 2a). Hence, the data suggests the following allelic series: *dAux*^*WT*^ > *dAux*^*RG*^ > *dAux*^*F956X*^ > *dAux*^*-*^.

To determine if the pathogenic mutation affects Auxilin protein levels over time, we conducted Western blotting of 5, 15 and 30-day-old (DO) adult head tissue of *dAux*^*WTWT*^, *dAux*^*RG/RG*^, *dAux*^*WT/F956X*^ and *dAux*^*RG/F956X*^ (the latter two only at 5 and 15DO). Interestingly, a decrease of Auxilin protein levels is observed over time, when compared to controls (Fig. 2b, c). Furthermore, the drop in protein levels is more pronounced when the R1119G and F956X mutations are combined compared to homozygous *dAux*^*RG/RG*^ (Fig. 2b, c). This further confirms that R1119G is a weak hypomorpic mutant and that it leads to a small age-dependent decline in protein abundance.

We next asked if the mutant *dAux* flies display progressive neurological problems, including motor defects and seizure-like behavior. In negative geotaxis assays, 5DO *dAux*^*RG/RG*^ flies can climb past a 4 cm mark within a 30 s window, however *dAux*^*RG/F956X*^ flies fail to do so (Fig. 2d, d’). Furthermore, this is progressive, and the ability to reach the 4 cm mark declines with age for both *dAux*^*RG/RG*^ and *dAux*^*RG/F956X*^ (Fig. 2d, d’ and Supplementary video 1–4). *DNAJC6/Auxilin* PD patients also frequently suffer from seizures. We therefore assessed this phenotype in 5DO flies triggered by 10 s of vortexing. This causes strong seizure-like behavior in both *dAux*^*RG/RG*^ and *dAux*^*RG/F956X*^ flies, but not in the controls (Fig. 2e, e’ and Supplementary video 5–8). These behavioral tests indicate that the pathological *DNAJC6/Auxilin* mutation causes neurological defects that are reminiscent of those observed in patients, including motor impairments and seizures.

### Auxilin R1119G causes neurodegeneration

**Fig. 3:**
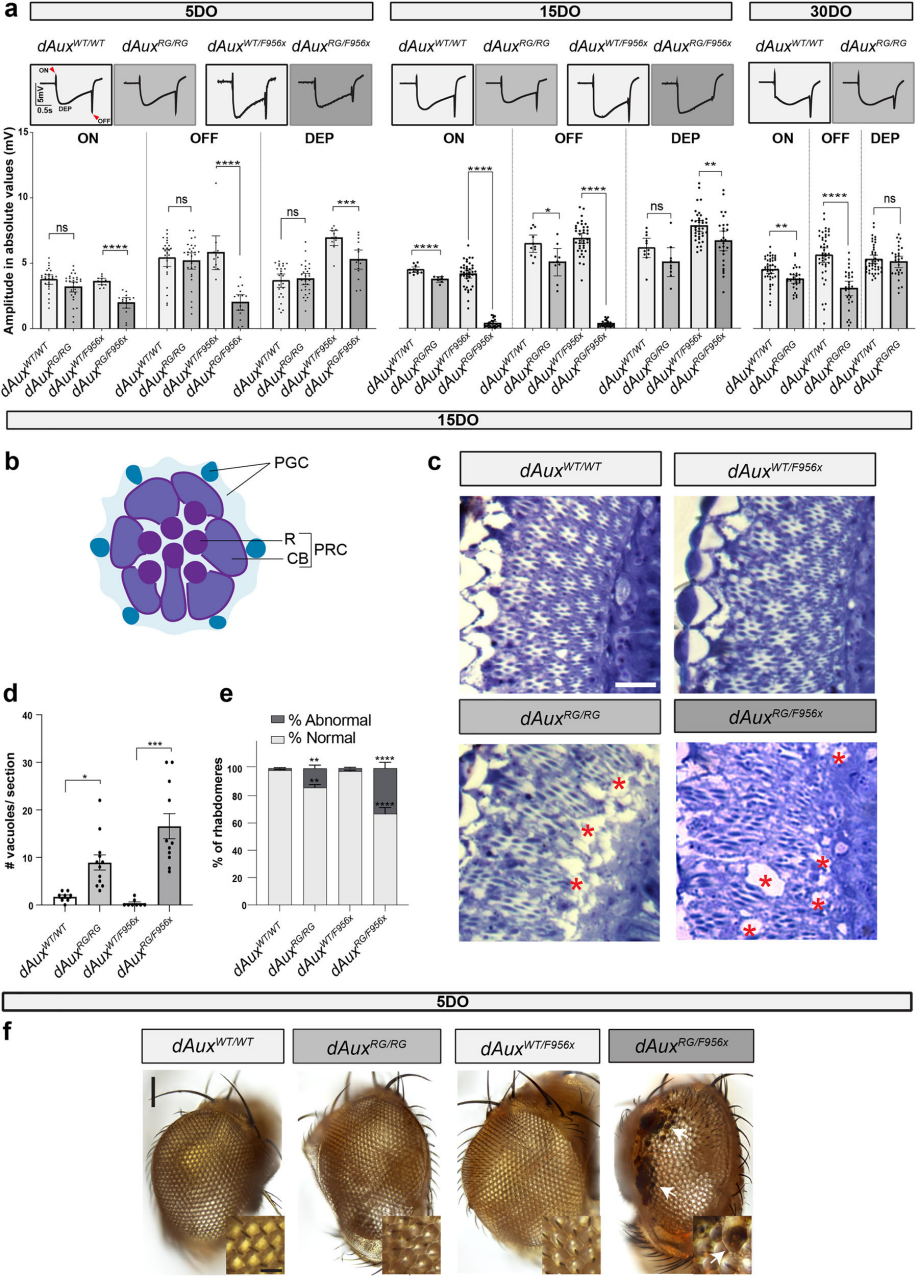
Pathogenic *Auxilin* mutants show neuronal function defects and neurodegeneration. **a** Average ERG traces of 5, 15 and 30DO flies of indicated genotypes and quantification on ON, OFF and depolarization amplitude. Arrowheads: “ON” and “OFF” transients. Bar graphs represent absolute mean ± SEM values (mV). n≥10 flies per genotype, points represent values obtained from individual flies. One-way ANOVA, Dunnett’s multiple comparison test. ns: not significant, **p < 0.01, ***p < 0.001 and ****p < 0.0001. **b** Diagram of a tangential section of the adult *Drosophila* retina, with pigmented glia cells (PGC) in blue and photoreceptor cells (PRC) in purple. R: rhabdomere; CB, cell body. **c**–**e** Representative brightfield images of toluidine blue stained sections of 15DO *dAux*^*WT/WT*^, *dAux*^*RG/RG*^, *dAux*^*WT/F956X*^ and *dAux*^*RG/F956X*^ fly eyes (**c**; asterisks indicate neurodegeneration) and quantification of the degenerated area (dubbed vacuoles in the literature/number of vacuoles counted) per standardized section (**d**) and the amount of normal *versus* abnormal rhabdomeres (see Methods) as a fraction of the total number of rhabdomeres (**e**). The graph in **d** shows the mean ± SEM of n≥10 flies per genotype from independent crosses and points are individual values. Kruskal-Wallis test, Dunnett’s multiple comparison test. *p < 0.05 and ***p < 0.001; the graph in **e** shows mean ± SEM of n≥5 flies per genotype from independent crosses. Two-way ANOVA with Tukey’s multiple comparison test. **p < 0.01 and ****p < 0.0001. Scale bar in **c** is 5 µm. **f** Representative brightfield images of the compound eye of 5DO flies of indicated genotypes. Insets show higher magnification. White arrows point to necrotic spots on the *dAux*^*RG/F956X*^ eye. Scale bars (up) 100 µm; (down) 20 µm.

To determine the integrity of neuronal function in adult flies, we performed electroretinogram (ERG) recordings. ERGs assess neuronal function and integrity of photoreceptors (the depolarization phase “DEP”) and synaptic transmission in the visual system (“ON” and “OFF” transients – red arrows) upon exposure to a short light pulse (1 s) (Fig. 3a)^19^. 5DO *dAux*^*RG/RG*^ flies show a stereotypical response, similar to *dAux*^*WT/WT*^ (Fig. 3a). However, 5DO *dAux*^*RG/F956X*^ flies show a lower DEP amplitude and smaller ON and OFF transients compared to controls (Fig. 3a). This phenotype worsens progressively, and 15 and 30DO animals show gradually smaller ON and OFF transient amplitudes (Fig. 3a). Finally, this defect is completely rescued by transgenic expression of wild-type *dAux* in neurons using *nSyb-Gal4* in 30DO animals (Supplementary Fig. 2a). These data indicate that the pathogenic mutation in Auxilin causes a cell-autonomous and progressive loss of neuronal integrity in mutant adult flies.

ON and OFF transient defects in ERG recordings arise from defects in synaptic transmission^19,52^, consistent with the known role of Auxilin in ensuring an efficient SV cycle^14,15,53^. The smaller depolarisation in ERG recordings suggests the *dAux* mutants cause neurodegeneration. We therefore prepared serial histological sections of 15DO animals and stained them with toluidine blue. Quantification revealed significantly more degeneration (“vacuoles”) in the optic lobes of *dAux*^*RG/RG*^ and *dAux*^*RG/F956X*^ flies compared to the controls (Fig. 3b–d)^54^. Furthermore, we also observe a loss of structural integrity in the compound eye. We find that 15DO *dAux*^*RG/RG*^ and *dAux*^*RG/F956X*^ animals have an increased number of abnormal ommatidia with fewer than 7 intact positioned rhabdomeres in a section (Fig. 3c, e). Also the external eye morphology is affected in 5DO *dAux*^*RG/F956X*^ animals, visible as a ‘rough eye phenotype’ and by the appearance of necrotic spots (Fig. 3f). Taken together, the pathogenic mutation in *DNAJC6/Auxilin* causes a loss of neuronal integrity and function, and of neurodegeneration.

### Pathogenic Auxilin mutations affect lipids that are critical to Synaptojanin function

**Fig. 4:**
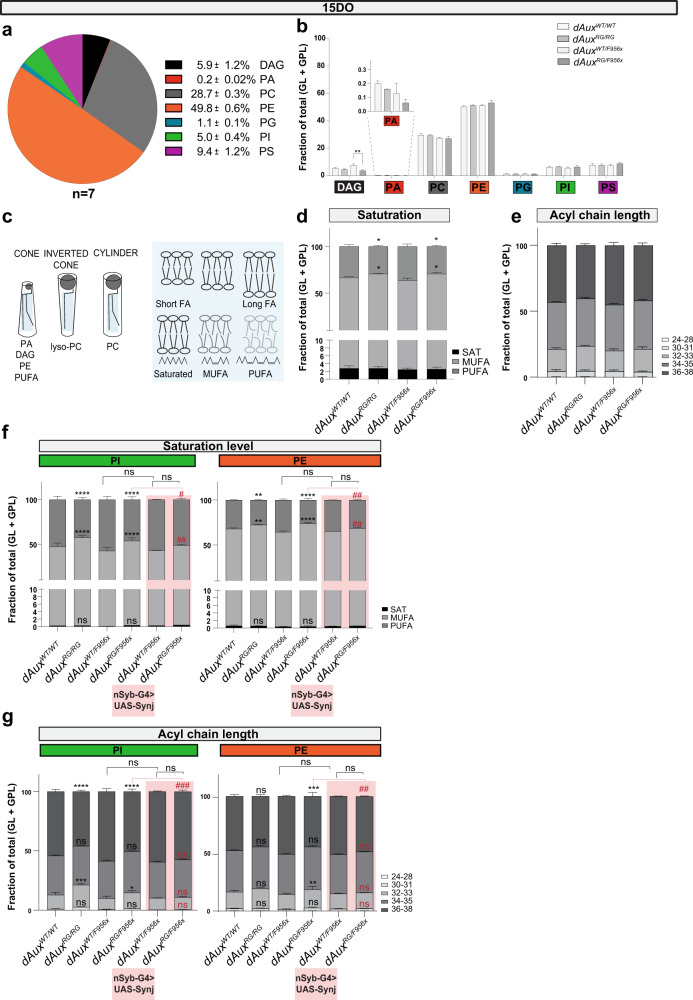
Membrane lipids containing Long-Chain Polyunsaturated Fatty Acids, including phosphoinositides species are decreased in pathogenic *Auxilin* fly heads. **a** Pie diagram of the relative abundance of individual GL and GPL classes in 15DO wild-type fly heads detected by mass spectrometry (MS). *n* = 7 analyses and one individual analysis is performed on 15 fly heads collected from 3 independent crosses. **b**. The relative abundance of individual GL and GPL classes in 15DO fly heads of indicated genotypes relative to *dAux*^*WT/WT*^ and *dAux*^*WT/F*^ as detected by mass spectrometry (MS). Bars show mean ± SEM of *n* ≥ 4 analyses. One individual analysis is performed on 15 fly heads collected from 3 independent crosses. Two-way ANOVA, Tukey’s multiple comparison test. **p < 0.01. **c** Schematic of the geometry of membrane lipids, showing the effect of polar headgroups and fatty acyl side chains. Membrane thickness, fluidity and deformity is influenced by the saturation level and length of the fatty acyl chain in concert with the presence of cholesterol^55^. **d**, **e** Saturation level (**d**) and length (**e**) of fatty acyl chains of all membrane lipids are presented as fraction of total membrane GL + GPL. Bars show mean ± SEM. n ≥ 4 and one individual analysis is performed on 15 fly heads. MUFA, mono-unsaturated; PUFA, poly-unsaturated. Two-way ANOVA, Tukey’s multiple comparison test. *p < 0.05. **f**, **g** Length (**f**) and saturation level (**g**) of fatty acyl chains of PI ad PE are presented as fraction of total membrane GL + GPL. Note the lower levels of LC-PUFAs including PI LC-PUFAs in *dAux* mutants. Plus, neuronal Synj expression partially rescues pathogenic *dAux*^*RG/F956x*^ mutant. Bars show mean ± SEM. n ≥ 4 analyses for *dAux*^*WT/WT*^, *dAux*^*RG/RG*^, *dAux*^*WT/F956x*^ and *dAux*^*RG/F956x*^ and *n* = 3 for *dAux*^*WT/F956x*^ and *dAux*^*RG/F956x*^ overexpressing Synj in neurons. One individual analysis is performed on 15 fly heads collected from 3 independent crosses. Two-way ANOVA, Tukey’s multiple comparison test. ns: not significant, *p < 0.05, **p < 0.01, ***p < 0.001 and ****p < 0.0001. # indicates comparison with *dAux*^*RG/F956x*^. ns: not significant, ^#^*p* < 0.05, ^##^*p* < 0.01 and ^###^*p* < 0.001.

Lipid disturbances are becoming increasingly prominent in PD^32–34^. It is conceivable that lipid alterations affect several cellular processes including vesicle budding mechanisms in the context of CME. To investigate if the pathogenic mutation in *DNAJC6/Auxilin* is associated with membrane lipid changes, we performed shotgun lipidomics of 15DO wild-type and mutant fly heads and examined membrane glycerol- and glycerophospholipids (GL and GPL). The major membrane GPLs we find in wild-type adult fly head are phosphatidylethanolamine (PE, ~50%), phosphatidylcholine (PC, ~29%), phosphatidylserine (PS, ~9%) and phosphatidylinositol (PI, ~5%) (Fig. 4a). The GPLs phosphatidic acid (PA) and phosphatidylglycerol (PG) and the GL diacylglycerol (DAG) are present in lower amounts (Fig. 4a). We find a similar relative abundance of GL and GPL in *dAux*^*RG/RG*^ and *dAux*^*RG/F956X*^ flies compared to the controls, except for the minor lipid species, DAG, that is reduced in *dAux*^*RG/F956X*^ mutants (Fig. 4b). This indicates that the composition of the major membrane glycerol and glycerophospholipids is not strongly affected by the pathogenic *Aux* mutation.

Membrane properties such as thickness, fluidity and curvature that affect synaptic function are dictated by lipid shape (e.g. cone- and inverted cone-shaped lipids – Fig. 4c^55^), but also by the fatty acid chain saturation level and length (Fig. 4c^55^). Furthermore, membrane lipids containing long-chain polyunsaturated fatty acids (LC-PUFAs) make up ~30% of the total brain lipids and are abundant in specialized organelles such as SVs^2,56,57^. Moreover, they are important for protein function and maintain a normal SV pool^36,58–61^. We therefore, closely examined fatty acid chain length and saturation level. When jointly examining all GLs and GPLs in 15DO *dAux*^*RG/RG*^ and *dAux*^*RG/F956X*^ mutant fly heads, we find an increase of mono-unsaturated fatty acids (MUFA) and a significant decrease in poly-unsaturated fatty acids (PUFA), compared to controls (Fig. 4d).

Next, we assessed fatty acid chain length and found a trend towards shorter chain length taking all membrane lipids (GL + GPL) together in *dAux*^*RG/RG*^ and *dAux*^*RG/F956X*^ mutants compared to controls (Fig. 4e). The lack of a significant change in fatty acid chain length in *dAux*^*RG/RG*^ and *dAux*^*RG/F956X*^ mutants could be masked since we analysed GL and GPL in bulk. We thus also investigated saturation level and chain length per lipid class. The most prominent change is a significant decrease in LC-PUFA PI species in both *dAux*^*RG/RG*^ and *dAux*^*RG/F956X*^ mutants compared to controls (Fig. 4f, g). More importantly, changes in PI 36:4:0, PI 36:3:0 and PI 32:1:0 levels are detected in both *dAux*^*RG/RG*^ and *dAux*^*RG/F956X*^ mutants (Supplementary Fig. 3a). Additionally, a significant decrease in PE species containing LC-PUFA is detected in *dAux*^*RG/F956X*^ mutants (Fig. 4f, g). This indicates that the pathogenic *DNAJC6/Auxilin* mutation induces changes in PI and PE lipid abundance and structure, including in lipid species that are profoundly required at synapses to regulate vesicle trafficking (LC-PUFA PI species).

### Neuronal Synaptojanin expression rescues pathogenic *DNAJC6/Auxilin* mutant defects

Membrane lipids containing LC-PUFA, PI and PE lipid species play a prominent role in neurons and at synapses^23,62,63^; several endocytic proteins interact with these lipids, including Synj1^35,36,61,64,65^. Given this evidence and that patients with mutations in *SYNJ1* show clinical phenotypes very similar to patients with mutations in *DNAJC6*^7,8,29–31^, we examined if the pathogenic *dAux*^*RG*^ mutation functionally interacts with *Synj*.

First, we examined the subcellular localization of Synj at the NMJ using anti-Synj and anti-Bruchpilot. Synj localizes to presynaptic nerve terminals^19^ as does Auxilin (Supplementary Fig. 4a and Fig. 1e), where it is adjacent to and partially overlaps with Bruchpilot. Synj is also present within the bouton lumen.

**Fig. 5:**
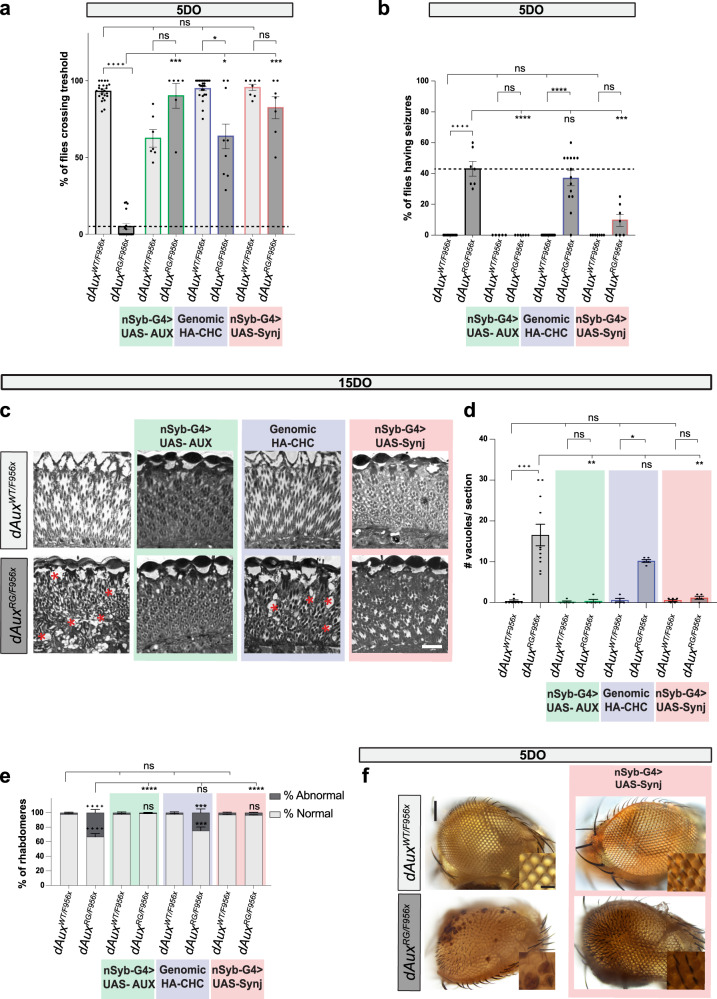
Neuronal expression of Synaptojanin rescues pathogenic Auxilin mutants. **a** Quantification of negative geotaxis assay of 5DO *dAux*^*WT/F956X*^ and *dAux*^*RG/F956X*^ flies and of *dAux*^*WT/F956X*^ and *dAux*^*RG/F956X*^ flies that neuronally express wild type *Drosophila* Auxilin (UAS-AUX), harbor an extra genomic copy of wild type *Drosophila* Clathrin heavy chain (HA-CHC) or neuronally express wild type *Drosophila* Synaptojanin (UAS-Synj). Graphs represent the fraction of flies crossing the threshold. Bars show mean ± SEM. Points represent the mean of three trials. One-way ANOVA, Dunnett’s multiple comparison test. ns: not significant, *p < 0.05, ***p *<* 0.001 and ****p < 0.0001. **b**. Quantification of seizures following stimulation in 5DO flies of indicated genotypes (see also **a**). Note that dAux and dSynj rescue pathogenic *dAux* mutants, but dChc does not. Graphs represent the fraction of flies that were unable to stand after 10 s after stimulation. Bars show mean ± SEM. Points represent single trials with 10 flies each. One-way ANOVA, Dunnett’s multiple comparison test, *n*≥5. ns: not significant, ***p < 0.001 and ****p < 0.0001. **c** - **e**. Brightfield images of toluidine blue stained sections of 15DO *dAux*^*WT/F956x*^ and *dAux*^*RG/F956X*^ flies neuronally expressing wild type *Drosophila* Auxilin (UAS-AUX), harboring an extra genomic copy of wild type *Drosophila* Clathrin heavy chain (HA-CHC) or neuronally expressing wild type *Drosophila* Synaptojanin (UAS-Synj) (**c**) and quantification of the degenerated area per standardized section (**d**) as well as the amount of normal *versus* abnormal rhabdomeres (see Methods) as a fraction of the total number of rhabdomeres (**e**). Note that again, dAux and dSynj rescue pathogenic *dAux* mutants, but dChc does not. Asterisks indicate degeneration in the retina. Scale bar: 5 µm. Graph in **d** shows mean ± SEM of n≥5 flies per genotype (from independent crosses), points represent individual values. Kruskal-Wallis test, Dunnett’s multiple comparison test. ns: not significant, *p < 0.05 and **p < 0.01. Graph in **e** shows mean ± SD of *n*≥5 flies per genotype from independent crosses. Two-way ANOVA with Tukey’s multiple comparison test. ns: not significant, **p < 0.01, ***p < 0.001 and ****p < 0.0001. **f**. Necrotic spots disappear and rough eye phenotype improves by neuronal expression of Synaptojanin. Panel shows representative brightfield images of the compound eye of 5DO flies of indicated genotypes. Insets show higher magnification. Scale bars (up) 100 µm; (down) 20 µm.

To determine more detailed whether the Auxilin R1119G mutation affects Synj localization we took advantage of the larval NMJ and the nanoscale resolution of the Airyscan detector. We quantified the amount of Synj present close to and at the synaptic plasma membrane and within the lumen of synaptic boutons by performing shell analysis. However, no significant alterations in Synj distribution were observed at this level of analysis (Supplementary Fig. 4b, b’). Next, Synj expression levels were determined by measuring the integrated intensity of labeling at the larval NMJ. This also revealed no significant differences in Synj levels between the different genotypes (Supplementary Fig. 4c, d). Given that a change in protein level or localization might be masked at this stage, we determined Synj protein levels in brain lysates of 15DO *dAux*^*WTWT*^, *dAux*^*RG/RG*^, *dAux*^*WT/F956X*^ and *dAux*^*RG/F956X*^. Interestingly, we find that at this adult stage, Synj protein levels were decreased in *dAux*^*RG/F956X*^ when compared to controls (Supplementary Fig. 4e, f). These results prompted us to conduct a genetic interaction experiment. We created *dAux*^*WT/F956X*^ and *dAux*^*RG/F956X*^ animals that overexpress Synj in neurons (using *nSyb-Gal4*). As an additional control we also created *dAux*^*WT/F956X*^ and *dAux*^*RG/F956X*^ animals that overexpress Aux in neurons. We find that overexpression of Synj in *dAux*^*RG/F956X*^ mutants is able to (partially) rescue the alterations in LC-PUFA PI species that were most prominently changed in *dAux*^*RG/RG*^ and *dAux*^*RG/F956X*^ mutants as well as in LC-PUFA PE species (Fig. 4f, g). Furthermore, Synj overexpression in neurons increased the levels of PI 36:4:0, PI 36:3:0 and reduced PI 32:1:0 restoring these lipid defects (Supplementary Fig. 3a). Interestingly, neuronal expression of Synj in *dAux* mutant flies not only rescues the negative geotaxis defects, it also prevents seizures to occur in 5DO mutant flies (Fig. 5a, b and Supplementary video 9–12), similarly to neuronal expression of wild type Auxilin. Additionally, neurodegeneration, measured by ERG and histology, of 15DO *dAux*^*RG/F956X*^ mutants is rescued (Fig. 5c–e and Supplementary Fig. 5a–d) and their external eye morphology is also markedly improved (Fig. 5f) by neuronal expression of Synj. In contrast, an additional genomic copy of the *Clathrin heavy chain* gene, another Auxilin-interacting protein that is not mutated in PD, fails to rescue most of these defects in *dAux*^*RG/F956X*^ mutants or only does so mildly (Fig. 5a–e and Supplementary Fig. 5a–d).

Taken together, these results indicate that Synj can compensate for the lipid, behavioral and functional defects and even prevent neurodegeneration induced by the pathogenic *Auxilin/DNAJC6* mutations.

## Discussion

In this work, we find a novel functional interaction between two genes mutated in Parkinsonism. We show that Synj levels decline in animals with a pathological *DNAJC6/Auxilin* mutation and that overexpression of the lipid phosphatase Synj, rescues the dysfunction induced by pathogenic mutations in *DNAJC6/Auxilin*. Auxilin regulates vesicle trafficking by helping to uncoat Clathrin from newly formed vesicles, including those budding from the trans-Golgi network and those forming at synaptic terminals. We show that *Aux* mutants induce specific lipid changes, including PI species containing LC-PUFAs, that are required for the localisation and function of Synj in endocytosis at synapses^35,36^. These findings suggest important roles for lipid metabolism in the pathogenicity of PD and they define a new functional group of proteins in Parkinsonism^7–10,27–31^, much like the discovery of the Pink1-Parkin pathway, where expression of *parkin* was shown to (partially) rescue aspects of *pink1* deficiency^66–68^.

There are several pathogenic mutations in *DNAJC6/Auxilin*; all share Parkinsonism phenotypes, but unlike the R927G (R1119G) mutation that we modelled here, other pathogenic mutations result in additional and more severe neurological defects^9,10,27,69^. Our data reveal that the R927G (R1119G) mutation is a weak loss-of-function mutation, in line with the relatively slow progression of the neurodegenerative features, as opposed to the very early onset neurological defects that are observed in the more severe loss-of-function mutations in *DNAJC6* (eg. Q734X and Q789X^9,69^).

A common feature across the *DNAJC6* mutations that cause Parkinsonism is that they affect the Auxilin J-domain (Fig. 1b). The J-domain is a co-chaperone domain that functions with Hsc-70 and the best-characterized function is its role in rearranging and dissolving the Clathrin basket to support uncoating of newly budded vesicles^13^. In the weak hypomorphic and pathogenic R927G (R1119G) *Aux* mutant animals, we did not find strong membrane uptake defects. However, we did observe smaller sized SVs that could be the result of inefficient Clathrin re-arrangements at the membrane. This may affect coated pit maturation, resulting in defective vesicle constriction and thus changes in SV size. Such Clathrin re-arrangement defects could equally affect other budding events in the cell like the formation of Golgi-derived vesicles. This is further supported by the fact that Auxilin is known to act at the Golgi^70,71^ and that dystrophic Golgi tubules were observed in Auxilin mutant mice^26^.

Lipid alterations are becoming increasingly prominent in Parkinson’s disease biology^32–34^. However, the functional consequences of these defects at the cellular and organellar level, and how they influence Parkinsonism progression, remains unclear. Our work suggests specific lipid changes to be central to synaptic and neuronal dysfunction in *DNAJC6/Auxilin*- and *Synj1*-induced Parkinsonism. We report that the R927G (R1119G) pathogenic mutant harbors lower amounts of GL and GPL containing LC-PUFA, including PI LC-PUFA species. Correctly positioned PIs and local PI metabolism are critical for numerous neuronal functions. Lipids containing LC-PUFAs are enriched at the nerve terminals and are highly abundant in SVs^2,60,72^. They regulate the progression of endocytosis and vesicle budding^35,61,64,65^, PI LC-PUFAs localize Synj1 and other proteins at synapses^35,61^, they activate the Sac1 domain phosphatase activity of Synj1^36^, they facilitate endosomal function^73^ and autophagosomal function^51^, etc. As such, even small lipid changes could have profound long-term effects and our working model is that the overexpression of Synj1 shifts the balance (back) such that more Synj1 can associate with membrane to exert its function at synapses^35,61,64,65^.

Our work also shows that not only the functional defects of *DNAJC6/Auxilin* mutants are restored by upregulation of Synj, but also many of the lipid changes. We surmise this could be because increased Synj levels help to recruit Auxilin towards its known function at the Golgi^70,71^. Most lipid metabolic enzymes operate at the ER, and this is the main center of GL and GPL generation, including lipids containing LC-PUFAs^74,75^. Lipids are then delivered to different locations through lipid transfer proteins and transport vesicles^74,76,77^. Hence, Golgi budding defects^26^ could contribute to the observed defects in lipid abundance in *dAux*^*RG*^ mutants.

Taken together, one model that is consistent with our data, is that pathogenic *DNAJC6/Auxilin* mutants act by affecting effective Clathrin uncoating in (1) Golgi vesicle budding, causing lipid delivery problems and (2) in SV production, causing functional problems. We thus envisage that overexpression of Synj compensates for the lower levels we observed in *dAux* mutants, and this could (1) drive Aux function at the Golgi to (partly) correct lipid levels and (2) overcome the lower PI PUFA abundance at synapses leading to enough Synj to associate with membranes to support synaptic function. However, our data does not exclude another mode of action, including that at synapses Auxilin, as a co-chaperone of HSC70, helps in the proper folding of Synj1 or maintains its stability, thereby fine-tuning its activity. Whether Auxilin affects Synj1 localization in adult flies remains to be demonstrated experimentally.

Our work is consistent with recent work on BioRxiv suggesting a role for pathogenic mutant *DNAJC6/Auxilin* at the Golgi^26^, and with synergistic genetic interactions between *DNAJC6/Auxilin* and Synj1^78^, thus defining a new functional module in Parkinsonism. Open questions for future work are to define how trafficking and lipid defects lead to degeneration, why some cells are more vulnerable to these defects than others, and whether similar lipid defects are also relevant in other (familial or idiopathic) forms of Parkinsonism.

## Methods

### Fly Stocks, maintenance, and tissue collection

*Drosophila* stocks (Supplementary Table 1) were maintained using standard protocols and fed a standard diet consisting of cornmeal, agar, yeast, sucrose, and dextrose. Experimental crosses were kept at 25 °C. L3 larvae of desired genotypes were selected to perform immunostaining, FM-43 dye uptake assays and TEM. To conduct behavioural assays, Western blotting, ERGs, toluidine blue staining to assess neurodegeneration and lipidomics, adult flies of desired genotypes were selected after eclosion and aged in groups of 10–20 flies for 5, 15, or 30 days at 25 °C. Flies were transferred to new vials every 3 days.

### New *Drosophila* Lines

Cloning was performed with the Gibson Assembly Master Mix (New England Biolabs). PCR products were produced with the Q5 High-Fidelity 2x Master Mix (New England Biolabs). Some fragments were ordered as gBlocks at IDT. All inserts were verified by sequencing. Microinjections were performed by BestGene Inc. (CA, USA). We used CRISPR-Cas9 to disrupt genomic dAux gene by injecting vas-cas9 (X chr) flies^79^ with pCFD3-dU6:3gRNA (Addgene #49410;^80^) carrying guide RNA sequences and a pWhiteStar donor plasmid carrying an integrase mediated cassette exchange (IMCE) and a *white* gene. Knock-ins were created by injecting pBS-KS-attB1-2 (Addgene#61255;^81^) containing HA-tagged *dAux*^*WT*^ or HA-tagged *dAux*^*RG*^ (the R1119G mutation) into recombinant vas-cas9:Phi31 flies. Transgenic fly lines were produced by injecting plasmids into VK37 for PhiC31 integrase-mediated site-specific transgenesis.

### HA-dAux^WT^ and HA-dAux^RG^

Unique gRNAs to disrupt the *dAux* gene were identified by flyCRISPR Target Finder Tool (https://flycrispr.org/) and predicted to introduce double-stranded breaks 249 bp and 222 bp before start and stop codon respectively. gRNAs were cloned into pCFD4:U6:1-gRNA U6:3-gRNA according to a previously established protocol^80^.

To generate a donor plasmid, DNA Assembly (NEB) was performed with four fragments: mini-*white* IMCE-cassette, left homology arm, right homology arm and pWhiteStar backbone^82^. Left and right homology arms (1 kb from gRNAs cutting side) of the *dAux* gene were ordered as gBlock at IDT. To avoid cutting the donor plasmid, the PAM sequences were mutated. The mini-*white* IMCE-cassette and vector backbone were generated by restriction digest on pWhite-STAR with AvrII and XhoI.

Next, a plasmid to exchange the mini-*white* IMCE-cassette was created by amplification of the entire *dAux* gene region from BAC CH322-22D05 (PACMANFLY^83^) with an N-terminal primer containing the HA-tag and cloning this into our pBS-KS-attB1-2 using BbsI. The R1119G mutation in *dAux* was produced by performing Q5® Site-Directed Mutagenesis (NEB).

### UAS-dAux

The coding sequence of *dAux* (NM_164317) was amplified from a pOT2 vector (DGRC, SD05837) and cloned between the EcoRI and XhoI sites of pUAST-attB^84^ using the Gibson NEBuilder® Hifi DNA assembly kit. The coding region of pUAST plasmids was fully verified by Sanger sequencing before further use. Detailed primer and gBlock information is provided in Supplementary Table 2.

### Behavioral assays

#### Negative geotaxis

Groups of 5 males and 5 female flies were transferred into a empty fly vial with a line at the height of 4 cm from the bottom of the vial. Flies were tapped down quickly and were then given 30 s to climb past the 4 cm mark. Three trials were conducted for each set of flies and the average percentage of flies crossing the mark across three trials was calculated and presented^85^.

#### Seizure assay

Seizure assays were performed with groups of 5 males and 5 female flies that were transferred into transparent vials and stimulmated by vortexing of the vial for 10 s a maximum intensity. Numbers of active and flies that were unable to stand were quantified 5 s later. Graphs are presented in % of flies having seizures^86^.

### Fluorescent labeling

#### Immunohistochemistry on larval NMJs

Larval preparations were fixed for 20 min at room temperature (RT) with 4% formaldehyde (Sigma #47608) in 1x phosphate buffered saline (PBS; pH 7.4), next larval fillets were permeabilized with 0.4% PBX (TritonX-100 in 1X PBS). Tissue was blocked for 1 h with 10% NGS in PBX and incubated overnight at 4 °C with primary antibodies. After several washes, larval fillets were incubated with secondary antibodies for 2 h in blocking solution at RT and washed with 0.4% PBX. Samples were mounted in Vectashield (Vector Laboratories). The following antibodies were used to label third instar *Drosophila* larvae: mouse α-Dlg [1:50 (DSHB; 4F3)], rabbit α-HRP [1:1000 (Jackson ImmunoResearch)], rabbit α-HA [1:200 (Cell Signaling Technologies; C29F4)], mouse α-Dynamin [1:50 (BD Biosciences, clone 41)], rabbit α-Synj [1:2000;^19^], mouse α-Brp [1:50 (DSHB; nc82)]. Alexa Fluor 488-/Alexa Fluor 555-conjugated secondary antibodies (Invitrogen) were used 1:1000.

#### Microscopy and image analysis

NMJs were imaged at muscles 12/13 in segment A2-A3 on a Nikon A1R confocal microscope with a Plan APO 60x A/1.20 Water Immersion DIC N2 lens using the NIS Elements software (Nikon). To quantify Auxilin and Synaptojanin expression levels, boutonic fluorescence intensities in maximum Z-projections were measured by applying the restrictive threshold “Moments” to determine ROIs/boutons after background subtraction. To determine more detailed the localisation of Synaptojanin within boutons, NMJs (at muscles 12/13 in segment A2-A3) were imaged using a Zeiss LSM 880 Airyscan microscope at 63× magnification (NA 1.4) in Airyscan mode. The Zen Black software (2012, Carl Zeiss) was used for acquisition and Airyscan processing. Next, we performed Shell Analysis on maximum Z-projections. Briefly, regions of interest were generated around the boutons and iterative shrinkage by a gap of 0.5 micron was applied to create two shells. The outermost shell is the periphery (shell 1) and the inner shell (shell 2) represents the lumen of the bouton. Then, the average integrated intensity of Synaptojanin was measured per shell. Peripheral signal was quantified by subtracting shell 2 from 1. Integrated intensities are represented as a fraction of total integrated intensity per bouton. Image acquisition and analysis was performed using ImageJ version 2.3.0 unless otherwise stated, by a researcher blind to genotype. At least three animals from independent crosses per genotype were examined in each analysis. Fly eyes were dissected and glued on a glass slide. For image acquisition, a Nikon NiE upright microscope was used in combination with a 10x Plan Apo lambda air objective (NA 0,45) and Nikon Fi3 color camera. The setup was controlled by NIS-Elements (NIS AR 5.41.01, Nikon Instruments Europe B.V.). Image stacks of about 230 micron were captured (z-step 2.5 micron) in brightfield using a combination of reflected and transmitted light illumination. Image stacks were transformed into a composite image via EDF processing (extended depth of field) using NIS-Elements.

### Transmission Electron Microscopy

Third instar larvae were dissected in fresh Ca^2+^ free HL3 (110 mM NaCl, 5 mM KCl, 10 mM NaHCO_3_, 5 mM Hepes, 30 mM sucrose, 5 mM trehalose, and 10 mM MgCl_2_, pH 7.2;^87^), nerves were cut and larvae subsequently incubated for 10 min in HL3 with 1.5 mM CaCl_2_ and 60 mM KCl. Multiple steps of washing with HL3 before fixation removed the non-internalized dye. Larvae were fixed in 4% paraformaldehyde (Laborimpex, 15714) and 2.5% glutaraldehyde (Polysciences, Inc, 111-30-8) in 0.1 M sodium-cacodylate buffer pH 7.4 (Merck, C0250-500g) at 4 °C for at least 24 h. The next day the larvae were osmicated in 2% osmium tetroxide (Laborimpex AGR1023) for 2 h on ice. After staining in 2% aqueous uranyl acetate solution (EMS #22400) for at least 1.5 h and dehydration in an ascending series of ethanol solutions, the samples were embedded in Agar 100 (Laborimpex, AGR1031) and cured at 60 °C for 48 h. Ultrathin sections (70 nm) were cut with a Dumont Diamond Knife on a Leica UCT ultra-microtome and collected on copper grids (Van Loenen, 01805-F) and imaged on a JEM 1400 transmission electron microscope (JEOL) at 80 kV with a bottom mounted camera (Quemasa; 11 megapixels; Olympus) running iTEM 5.2 software (Olympus). Statistical analyses were performed on larvae from 3 independent crosses per genotype. More than 5 NMJs per larvae were imaged.

### Machine learning to detect synaptic vesicles at NMJ

Electron micrographs were first denoised using ImageJ and Tikhonov algorithm using following parameters; lambda = 2, iterations = 5, and sigma = 1.058. Fifty-five random cropped denoised EM images of 512 by 512 pixels were manually annotated using QuPath^88^ and exported as labelled images using a custom Script. Cropped images and associated labels have been used to train a new model using StarDist and exported to be used with the StarDist ImageJ plugin^89^. A lower probability threshold was used to detect all SVs inside a given bouton. The SVs detected are highlighted as ROIs from which total amount and diameter were quantified. Three random tiles of 500 by 500 nm per bouton were examined. More than four boutons per animal per genotype were imaged.

### Toluidine blue staining

Adult fly heads were decapitated and immediately fixed in 4% paraformaldehyde (Laborimpex, 15714) and 2% glutaraldehyde (Polysciences, Inc, 111-30-8) in 0.1 M Na-Cacodylate buffer pH 7.4 (Merck, C0250-500g) for 2 h at RT. Samples were further fixed at 4 °C overnight, and then washed with 0.1 M Na-Cacodylate, pH 7.4, and subsequently osmicated with 2% osmium tetroxide (Laborimpex AGR1023). After staining in 2% uranyl acetate solution (EMS #22400) for at least 1.5 h and dehydration in an ascending series of ethanol solutions, samples were embedded in Agar 100 (Laborimpex, AGR1031) and cured at 60 °C for 48 h. Alternatively, the sampes were kept substantially longer in 2% osmium tetroxide and kept overnight in 0.5% Uranylacetate/25% methanol solution before continuing with the dehydratation and embedding steps. Semi thin sections (1.5 µm) of the fly heads were collected on microscopy slides. The sections were then dried and stained on a heating block with a 1% toluidine blue (Merck, 89640-5 G) solution including 2% Borax for 90 s at 60 °C. The stained sections were mounted with Eukit Quick-hardening mounting medium (Sigma Aldrich, 03989-500 ML) and imaged with the Leica DM25000M and 20x and 63x objectives. The percentage of normal vs abnormal ommatidia (defined as altered number and/or position of rhabdomeres) and the number of vacuoles in the fly retina were manually quantified using ImageJ.

### FM 1-43 dye uptake assays

Labelling of FM1-43 and quantification of intensities was performed as previously described^19^. Briefly, third instar larvae were dissected in fresh Ca^2+^ free HL3^87^, nerves were cut and subsequently incubated for 10 min in HL3 with 4 µM FM 1-43 (Invitrogen), 1.5 mM CaCl_2_ and 90 mM KCl. Multiple steps of washing with HL3 before imaging removed the non-internalized dye. NMJs were imaged at muscles 12/13 in segment A3 on a Nikon A1R confocal microscope through a 60X, 1.0 NA water immersion lens and stored using NIS elements software package. Mean boutonic intensities were determined, after background subtraction, using ImageJ.

### Electroretinograms

Electroretinograms (ERGs) were recorded as previously described^90^. Briefly, flies were immobilized on glass microscope slides by use of 5 s fix UV glue. For recordings, glass electrodes (borosilicate, 1.5 mm outer diameter) filled with 3 M NaCl were placed in the thorax as a reference and on the fly eye for recordings. Responses to repetitive light stimuli were recorded using Axosope 10.7 and analyzed using Clampfit 10.7 software (Molecular Devices) and Igor Pro 6.37.

### Western Blotting

Fly heads were obtained from 3 independent crosses through flash freezing with liquid nitrogen, followed by vortexing and sieving to separate heads from bodies. Homogenization of samples was obtained using a motorized pestle and 100 µL T-PER (Thermo Fischer Scientific) containing protease inhibitors (Sigma). 20/30 µg of protein was subjected to standard SDS-PAGE and Western blotting with HFP-conjugated secondary antibodies. The HRP-signal was visualized after incubation with the West Pico Plus chemiluminescent reagent (Thermo Fischer Scientific)) or the Western Lightning™ Plus Chemiluminescence Reagent (Perkin Elmer) using the iBright imaging system (Thermo Fisher Scientific). The following antibodies were used: rabbit anti-GAPDH [1:2000 (Abcam)], rabbit anti-DNAJC6 [1:1000 (Pierce)], rabbit α-Synj [1:2000;^19^], rabbit anti-GAPDH [1:10000 (Invitrogen)] and HRP-conjugated anti-rabbit seconday antibodies [1:5000 (Jackson ImmunoResearch)].

### Lipidomic mass spectrometry

Mass spectrometry was performed on heads of 15DO flies homogenized in 300 µl D-PBS (Dulbecco’s phosphate-buffered saline without Mg^2+^ and Ca^2+^) by Lipotype. Fifteen fly heads were pooled from three independent crosses for each analysis, and mass spectrometry was performed on n = 7 for wild-type, n ≥ 4 analyses for *dAux*^*WT/WT*^, *dAux*^*RG/RG*^, *dAux*^*WT/F956x*^ and *dAux*^*RG/F956x*^ and *n* = 3 for *dAux*^*WT/F956x*^ and *dAux*^*RG/F956x*^ overexpressing Synj. All liquid handling steps were performed using Hamilton Robotics STARlet robotic platform featuring the Anti Droplet Control for improved organic solvents handling. Prior to lipid extraction using chloroform and methanol, samples were spiked with lipid class-specific internal standards. After drying and resuspending in an appropriate MS acquisition mixture, lipid extracts were infused directly in QExactive mass spectrometer (Thermo Fisher Scientific) with TriVersa NanoMate ion source (Advion Biosciences). Samples are analyzed in both positive and negative ion modes, with MS resolution R_m/z = 200_ = 280000 and MSMS resolution R_m/z = 200_ = 17500, in a single acquisition. Acquired data was processed using lipid identification software based on LipidXplorer^91^. Data post-processing and normalization were performed by Lipotype using a developed data management system. Data analysis was performed using GraphPad Prism software 9.3.1. Pmol values (obtained by MS) of individual lipid species were transformed into a fraction of the total PA, DAG, PI, PC, PE, and PS lipids in the sample.

### Statistical analyses

Statistical analyses were performed using GraphPad Prism 9.3.1 software. Data is presented as mean ± SEM, unless otherwise stated. Differences between two groups were assessed using a two-tailed Student’s *t*-test, differences between two or more variables were assessed by one-way or two-way ANOVA, with Tukey’s or Dunnett’s multiple correction test, unless otherwise stated. The criteria for significance are: ns (not significant), *p < 0.05, **p < 0.01, ***p < 0.001, ****p < 0.0001. Comparison with *dAux*^*RG/F956x*^ is indicated with an #. ns: not significant, ^#^*p* < 0.05, ^##^*p* < 0.01 and ^###^*p* < 0.001.

### Reporting summary

Further information on research design is available in the Nature Research Reporting Summary linked to this article.

## Supplementary information

Supplementary information

Unprocessed WB scans

Reporting Summary

Supplementary video 1_dAuxWTWT_neggeo_15DO

Supplementary video 2_dAuxRGRG_neggeo_15DO

Supplementary video 3_dAuxWTF956x_neggeo_15DO

Supplementary video 4_dAuxRGF956x_neggeo_15DO

Supplementary video 5_dAuxWTWT_seizures_5DO

Supplementary video 6_dAuxRGRG_seizures_5DO

Supplementary video 7_dAuxWTF956x_seizures_5DO

Supplementary video 8_dAuxRGF956x_seizures_5DO

Supplementary video 9_dAuxWTF956x_SynjCTRL_neggeo_5DO

Supplementary video 10_dAuxRGF956x_SynjCTRL_neggeo_5DO

Supplementary video 11_dAuxWTF956x_SynjCTRL_seizures_5DO

Supplementary video 12_dAuxRGF956x_SynjCTRL_seizures 5DO

## Data Availability

Detailed information is provided in tables for *Drosophila melanogaster* lines (Supplementary Table 1), primers and gBlocks (Supplementary Table 2) used in this study. The lipidomic data generated by Lipotype is available at the NIH Common Fund’s National Metabolomics Data Repository, the Metabolomics Workbench, https://www.metabolomicsworkbench.org where it has been assigned Project ID PR001498. The data can be accessed via the Project 10.21228/M83D9C. This database is supported by the NIH grant *U2C-DK119886*.
