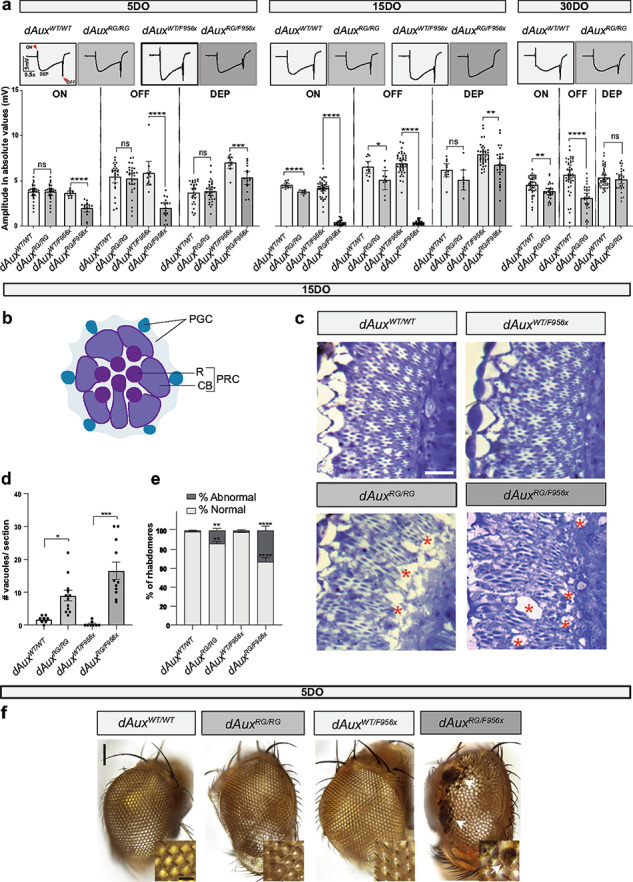# Author Correction: Parkinsonism mutations in *DNAJC6* cause lipid defects and neurodegeneration that are rescued by Synj1

**DOI:** 10.1038/s41531-026-01327-6

**Published:** 2026-04-02

**Authors:** Julie Jacquemyn, Sabine Kuenen, Jef Swerts, Benjamin Pavie, Vinoy Vijayan, Ayse Kilic, Dries Chabot, Yu-Chun Wang, Nils Schoovaerts, Nikky Corthout, Patrik Verstreken

**Affiliations:** 1 VIB-KU Leuven Center for Brain & Disease Research, Leuven, Belgium; 2 KU Leuven, Department of Neurosciences, Leuven Brain Institute, Mission Lucidity, Leuven, Belgium; 3 VIB-Bioimaging Core, Leuven, Belgium; 4 Present Address: Neuroscience and Mental Health Institute, University of Alberta, Department of Physiology, Department of Cell Biology, Group on Molecular and Cell Biology of Lipids, Edmonton, Alberta Canada; 5 Present Address: VIB Technology Watch, Technology Innovation Laboratory, VIB, Gent, Belgium

Correction to: *npj Parkinson’s Disease* 10.1038/s41531-023-00459-3, published online 04 February 2023

In this article Fig 3a appeared incorrect; the data points in GraphPad for plotting the bar graphs of dAuxwt/wt and dAuxRG/RGERG on amplitude were the same and have now been corrected in the original publication. For completeness and transparency, the old incorrect version is displayed below.