# Supplementary material for: Parkinsonism mutations in DNAJC6 cause lipid defects and neurodegeneration that are rescued by Synj1
Source: NPJ Parkinsons Dis. 2023 Feb 4;9:19. doi: 10.1038/s41531-023-00459-3 (PMC9899244; doi:10.1038/s41531-023-00459-3)
Supplement: Supplementary file 1 — Supplementary information [file 41531_2023_459_MOESM1_ESM.pdf]

Supplementary information

Parkinsonism mutations in *DNAJC6* cause lipid defects and neurodegeneration that are rescued by Synj1

Julie Jacquemyn[1,2,*, §], Sabine Kuenen[1,2,*], Jef Swerts[1,2], Benjamin Pavie[1,2,3] , Vinoy Vijayan[1,2], Ayse Kilic[1,2], Dries Chabot[1,2], Yu-Chun Wang[1,2,&], Nils Schoovaerts[1,2], Nikky Corthout[1,2,3] and Patrik Verstreken[1,2,#]

[1] VIB-KU Leuven Center for Brain & Disease Research, 3000 Leuven, Belgium
[2] KU Leuven, Department of Neurosciences, Leuven Brain Institute, Mission Lucidity, 3000 Leuven, Belgium
[3] VIB-Bioimaging Core, 3000 Leuven, Belgium

* Equal contribution
[§] Present address: Neuroscience and Mental Health Institute, University of Alberta, Department of Physiology, Department of Cell Biology, Group on Molecular and Cell Biology of Lipids of Dr. Maria S. Ioannou, Edmonton, Alberta, Canada
[&] Present address: VIB Technology Watch, Technology Innovation Laboratory, VIB, Gent, Belgium

[#] Correspondence: patrik.verstreken@kuleuven.be

Table of Contents

Supplementary Figure 1. dAux$^{RG/RG}$ or dAux$^{RG/F956x}$ mutations do not obviously affect synaptic vesicle endocytosis at larval NMJs 3

Supplementary Figure 2. Neuronal expression of wild-type dAux rescues neuronal defects in 30DO dAux$^{RG/RG}$ flies 4

Supplementary Figure 3. Auxilin mutant flies show alterations in PI species that are rescued by neuronal expression of Synj 5

Supplementary Figure 4. Synaptojanin levels are lower in 15DO dAux$^{RG/F956X}$ flies 6

Supplementary Figure 5. Expression of wild type Drosophila Synaptojanin restores neuronal function in Auxilin mutants 8

Supplementary Table 1. Drosophila stocks 10

Supplementary Table 2. Primers and gBlocks 11

Supplementary video 1 – 4: Negative geotaxis at 15DO of dAux^{WT} , dAux^{RG}, dAux$^{WT/F956x}$ and dAux$^{RG/F956x}$. 14

Supplementary video 5 – 8: Seizure assay at 5DO of dAux^{WT} , dAux^{RG}, dAux$^{WT/F956x}$ and dAux$^{RG/F956x}$. 14

Supplementary video 9 – 10: Negative geotaxis at 5DO of dAux$^{WT/F956x}$ and dAux$^{RG/F956x}$ overexpressing Synj in neurons. 14

Supplementary video 11-12: Seizure assay at 5DO of dAux$^{WT/F956x}$ and dAux$^{RG/F956x}$ overexpressing Synj in neurons. 14

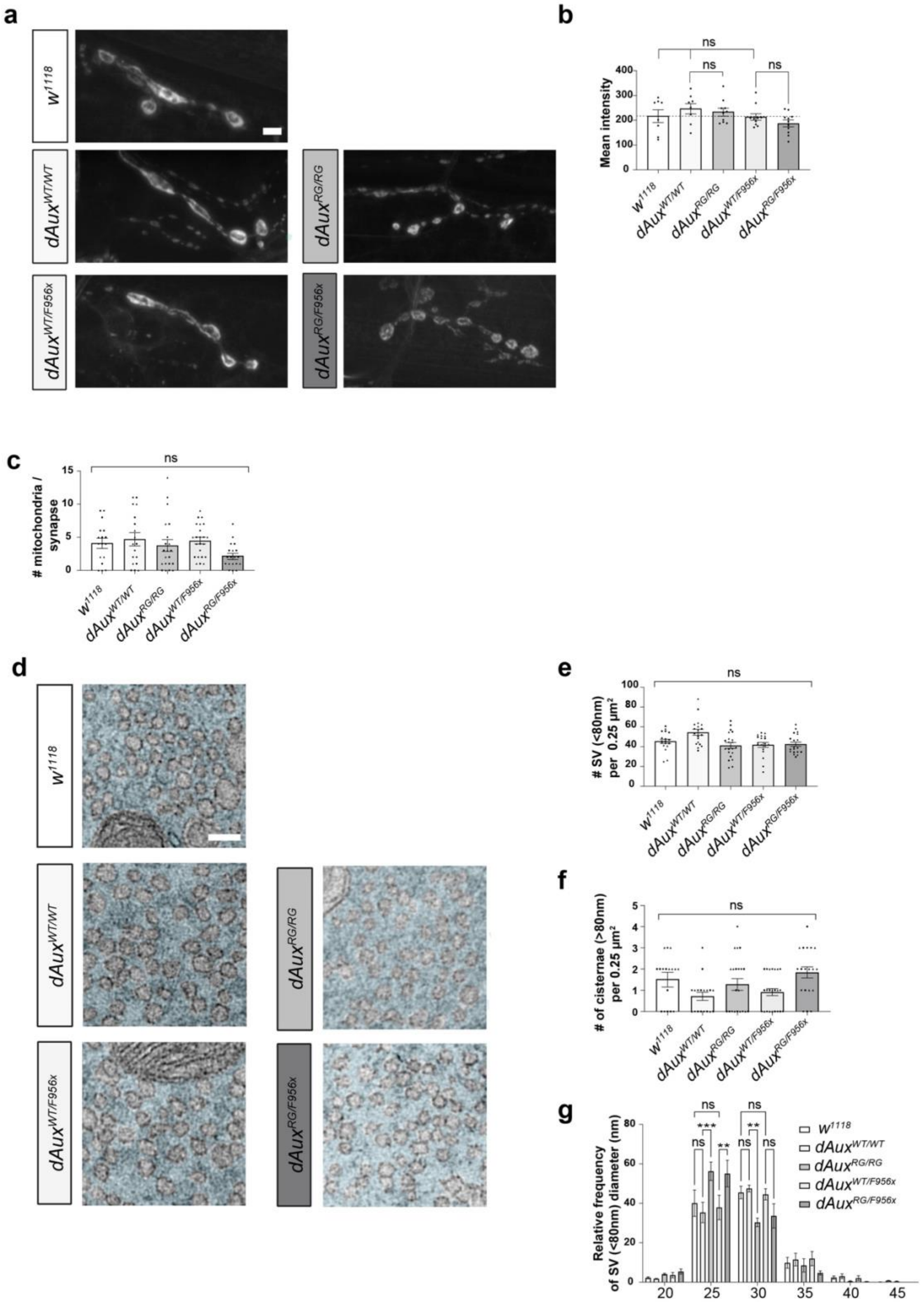
a
b
c
d
e
f
g
ns
Mean intensity
mitochondria / synapse
SV (<80nm) per 0.25 µm²
# of cisternae (>80nm) per 0.25 µm²
Relative frequency of SV (<80nm) diameter (nm)
w1118
dAuxWT/WT
dAuxRG/RG
dAuxWT/F956x
dAuxRG/F956x

Supplementary Figure 1. *dAux*$^{RG/RG}$ or *dAux*$^{RG/F956x}$ mutations do not obviously affect synaptic vesicle endocytosis at larval NMJs

a. Representative images of larval NMJs loaded (10 min, HL3 with 1.5 mM $CaCl_2$ and 90 mM KCl) in the presence of the lipophilic dye FM1-43 of the following genotypes: w^{1118}, *dAux*$^{WT/WT}$, *dAux*$^{RG/RG}$, *dAux*$^{WT/F956x}$ and *dAux*$^{RG/F956x}$.

b. Quantification of the mean FM1-43 intensity. Bars show mean ± SEM. Dotted line indicates mean of w^{1118}. Dots represent mean intensity obtained from 5 NMJs per larvae. n≥8 L3 larvae (from independent crosses) per genotype were analysed. Bars show mean ± SEM. Kruskal-Wallis test with Dunnett's multiple comparison test. ns: not significant.

c. Quantification of the number of mitochondria inside each synapse. Bars show mean ± SEM. Dots represent the number of mitochondria per bouton and different shapes indicate the boutons belonging to the same animal. Kruskal-Wallis test with Dunnett's multiple comparison test. ns: not significant.

d. Synapse ultrastructure of NMJs of w^{1118}, *dAux*$^{WT/WT}$, *dAux*$^{RG/RG}$, *dAux*$^{WT/F956x}$ and *dAux*$^{RG/F956x}$ larvae. Prior to TEM, larval fillets were incubated for 10 min in HL3 with 1.5 mM $CaCl_2$ and 60 mM KCl. Scale bar indicates 100 nm.

e & f. Graphs represent the density of synaptic vesicles with a diameter below or above 80nm. Three random tiles of 500 by 500 nm (0.25 μm^2) per bouton were analysed by applying a machine learning algorithm (see methods). More than four boutons per animal and three animals per genotype were examined. Dots represent the average density calculated and different shapes indicate the boutons belonging to the same animal. Bars show the mean ± SEM. Kruskal-Wallis test with Dunnett's multiple comparison test. ns: not significant.

g. Synaptic vesicle diameter is shifted to lower values in boutons of *dAux*$^{RG/RG}$ and *dAux*$^{RG/F956x}$ mutants compared to their proper controls. Diameters of SV were measured as mentioned in **d** & **e** and the relative frequency distribution of the diameter in bins of five was calculated. Bars show the mean ± SEM. Two-way ANOVA with Tukey's multiple comparison test. ns: not significant, ** $p < 0.01$ and *** $p < 0.001$.

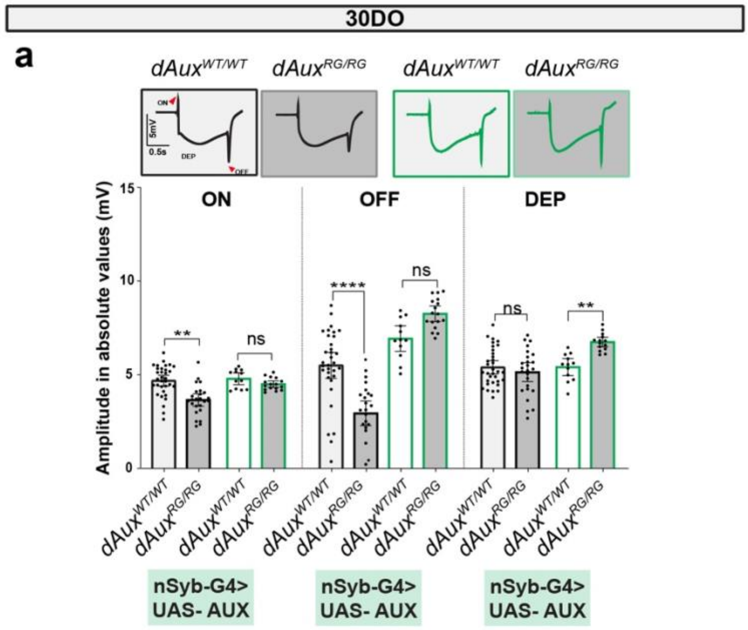

Supplementary Figure 2. Neuronal expression of wild-type *dAux* rescues neuronal defects in 30DO *dAux*$^{RG/RG}$ flies

a. Average ERG traces of 30DO *dAux*$^{WT/WT}$ and *dAux*$^{RG/RG}$ flies neuronally expressing Auxilin using the Nsyb-Gal4 driver. “ON” and “OFF” peaks are indicated with arrowheads. The ERG ON/OFF transient and amplitude of depolarization (DEP) are quantified and represented as absolute values (mV) in the graphs below. Graphs represent the mean ± SEM of n≥10 per genotype, dots represent individual values. One-way ANOVA, Dunnett’s multiple comparison test. ns: not significant, ** $p < 0.01$ and **** $p < 0.0001$.

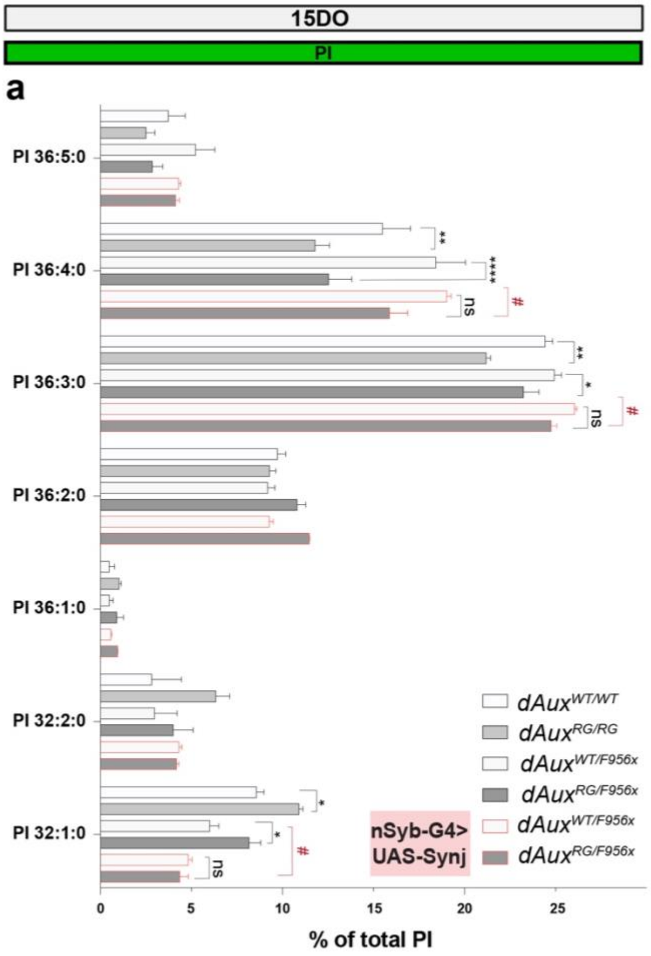

Supplementary Figure 3. *Auxilin* mutant flies show alterations in PI species that are rescued by neuronal expression of Synj

a. The abundance of individual PI species are presented as % of total PI. Bars show mean ± SEM. n≥4 Analyses for *dAux*$^{WT/WT}$, *dAux*$^{RG/RG}$, *dAux*$^{WT/F956x}$ and *dAux*$^{RG/F956x}$ and n=3 for *dAux*$^{WT/F956x}$ and *dAux*$^{RG/F956x}$ overexpressing Synj. One individual analysis is performed on 15 fly heads collected from 3 independent crosses. Two-way ANOVA, Tukey's multiple comparison test. ns: not significant, * $p < 0.05$, ** $p < 0.01$ and **** $p < 0.0001$. # indicates comparison with *dAux*$^{RG/F956x}$. ns: not significant and $^{\#}$ $p < 0.05$.

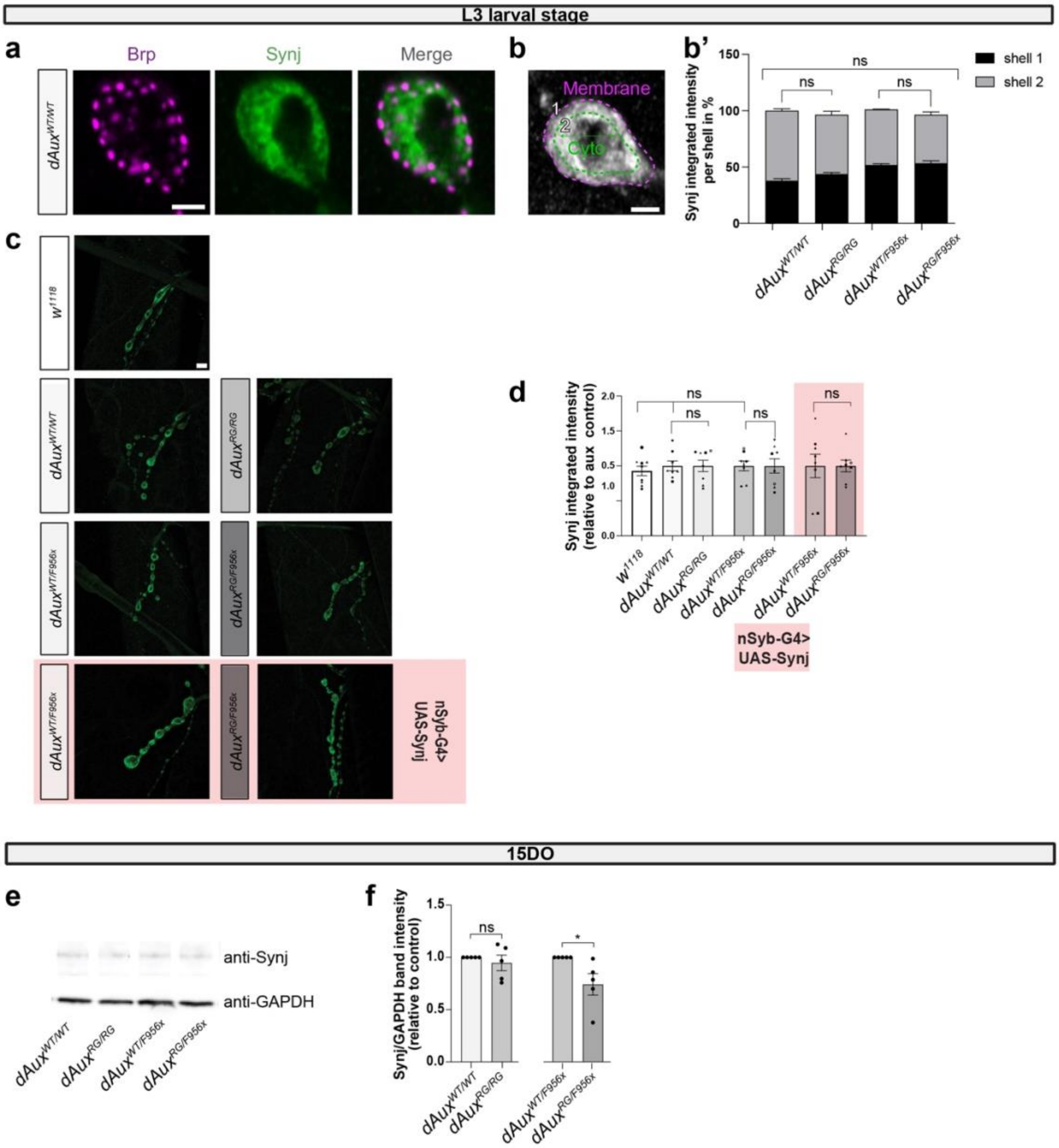

Supplementary Figure 4. Synaptojanin levels are lower in 15DO *dAux*$^{RG/F956X}$ flies

a. Synaptojanin localizes to the nerve terminal of larval NMJs where it is adjacent to and partially overlaps with the active zone protein Bruchpilot. Synaptojanin is also present within the lumen of the bouton. Representative Airyscan image of NMJ labelled with anti-Synaptojanin (green) and anti-Bruchpilot (magenta) in *dAux*$^{WT/WT}$. Scale bar: 2 µm.

b & b'. Quantification of Synaptojanin intensity at the plasma membrane (magenta) and within the synaptic lumen (green) using shell analysis (**b**). See Methods for more details. Quantification of the Synaptojanin integrated intensity across genotypes indicated in (**b'**) showing intensity at membrane (shell 1) and lumen (shell 2). Bars show mean ± SEM. n=3 L3 larvae (from independent crosses) per genotype were analysed. Two-way ANOVA, with Sidak's multiple comparison test. ns: not significant.

c & d. Representative images of NMJs labelled with anti-Synaptojanin (**c**). Quantification of the mean integrated intensity of Synaptojanin per NMJ (**d**). Dots represent mean integrated intensity obtained from 3 NMJs per larvae. n≥8 L3 larvae (from independent crosses) per genotype were analysed. Bars show mean ± SEM. Kruskal-Wallis test with Dunnett's multiple comparison test. ns: not significant. Scale bar: 5 µm.

e & f. Images of Western blots from brain lysates prepared from 15DO flies of indicated genotypes labelled with anti-Synaptojanin and anti-GAPDH (loading control) (**e**) and quantification of Synaptojanin protein levels (**f**). Values are relative to GAPDH and are expressed as a fraction of protein levels in $dAux^{WT/WT}$ and $dAux^{WT/F956x}$. Bars show the mean ± SEM, points show individual values and n≥2. Kruskal-Wallis test with Dunn's multiple comparison. ns: not significant and *$p<0.05$.

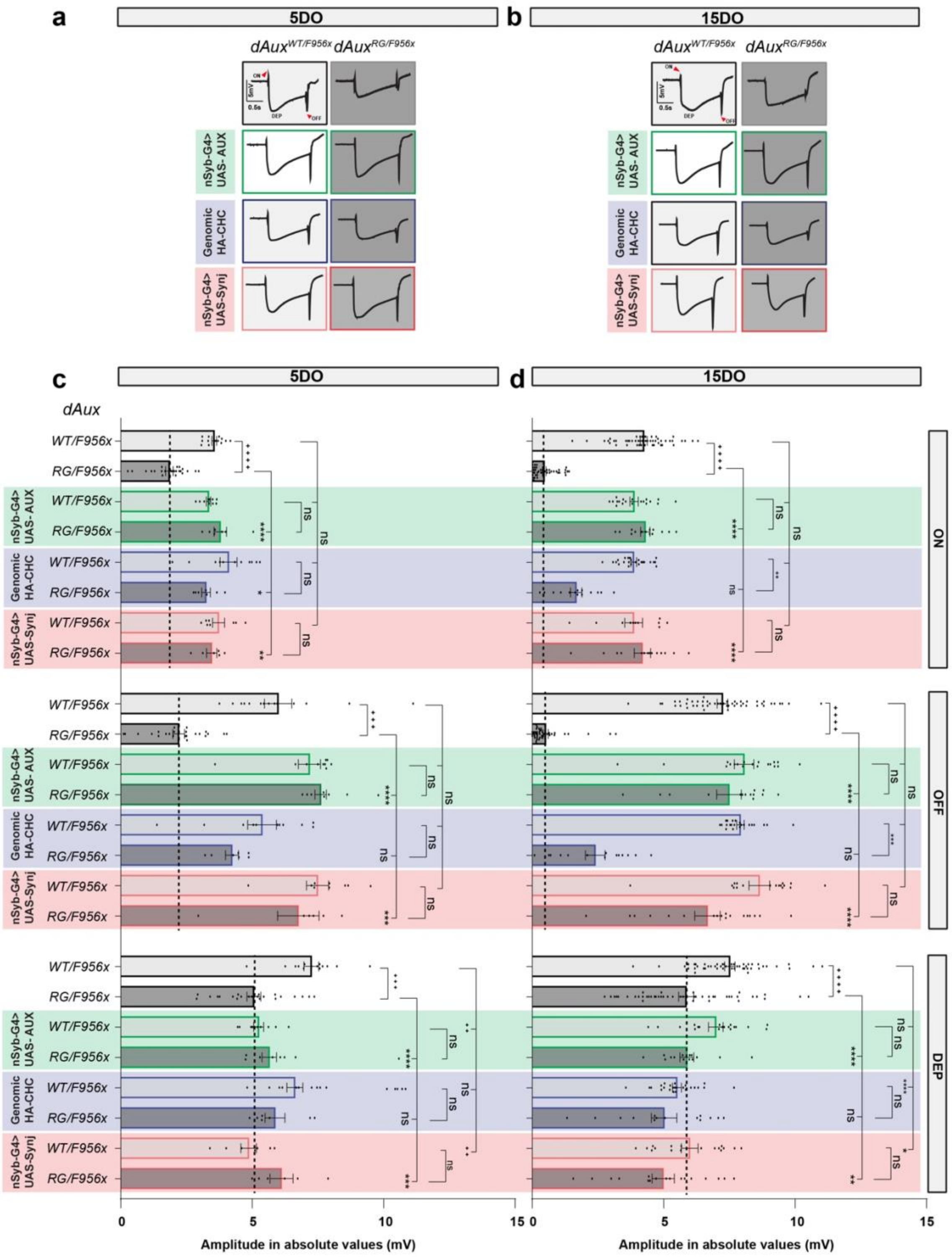

Supplementary Figure 5. Expression of wild type *Drosophila* Synaptojanin restores neuronal function in Auxilin mutants

a & b. Average ERG traces of 5 and 15DO *dAux*$^{WT/F956x}$ and *dAux*$^{RG/F956x}$ flies that neuronally express wild type *Drosophila* Auxilin (UAS-AUX), harbor an extra genomic copy of wild type

Drosophila Clathrin heavy chain (HA-CHC) or neuronally express wild type *Drosophila* Synaptojanin (UAS-Synj).

c & d. Quantification of the ERGs of 5DO (**c**) and 15DO (**d**) *dAux*$^{WT/F956x}$ and *dAux*$^{RG/F956x}$ flies that express wild type *Drosophila* Auxilin (UAS-AUX), harbor an extra genomic copy of wild type *Drosophila* Clathrin heavy chain (HA-CHC) or neuronally express wild type *Drosophila* Synaptojanin (UAS-Synj).The ERG ON/OFF transient and amplitude of depolarization are quantified and represented as absolute values (mV). Graphs show mean ± SEM of n≥5 per genotype, dots represent individual values. One-way ANOVA, Dunnett's multiple comparison test. ns: not significant, * $p < 0.05$, ** $p < 0.01$, *** $p < 0.001$, **** $p < 0.0001$.

Supplementary Table 1. *Drosophila* stocks

Line name	Genotype	Stock number	Reference (if relevant)
Genomic insertions			
HA-*dAux*^{RG}	w^{-};; HA-*dAux*^{RG}/TM6c,Tb		This study
HA-*dAux*^{WT}	w^{-};; HA-*dAux*^{WT}/TM6b,Tb,Hu		This study
Mutant line created by EMS			
dAux^{F956x}	w^{-};; dAux^{F956x}/Tm6b,Tb,Hu		Gift of Dr. Henry Chang, Purdue University
HA-CHC	w^{-}; HA-CHC/CyO		Generated previously in the lab [79]
UAS-lines			
UAS-dAux	w^{-};; UAS-dAux/CyO,Tb		This study
UAS-dSynj	w^{-}; UAS-dSynj/CyO,Tb		Generated previously in the lab [51]
Drivers			
nSyb(57C10)-GAL4		BDSC (39171)	

Supplementary Table 2. Primers and gBlocks

Plasmids	Fragment amplification
pCFD4:U6:1-gRNA1 U6:3-gRNA2	5’ aaacGCTCTTATCGATGTGGCGCG 3’/<br>5’ cttcCACCATGGTGACACCGACGG 3’
pWhite-Star	Left homology arm (gBlock):<br>5’ GGTACCTACCTAGGATATGCAATGCTAGATCGGGGGCATCGATACAACGTGGGAGTAGGGCGGTGGTAATTAGTACACTGATAAGGTTGTGTATTTTGCATTCGACAAATCTCCACGCGTTACGTGAAACTGGCCAGGTGCACACAGCAAATTGCAAGCTTAACTTCAATGTCCAAAATAAGCGGAGCTCGGTTTGCGCAAACTTACGGCCATATCGGTCATCTGTAACTATCCGTATTCGCCAGCGGCCCAAGCGTAGCGCAACACAGCTCTCAATTATCCAAAGACAGATGTGTAAATATACTGCCGTTACCAACTTGTTGCTGTTCCACGGAGGGGAGGTGCCCCAAAGGCTTACTAGTCATACTCCATTTCTTCATTCCGACGGCCAGCTGTTGCAGCGGTTGTCCCCTGGGTTTCCCCTTAGCTGTTGACTTCAATCAGCCAACTCTACCTGGCACAATGTACATAGGAAACTCAAACTTCAGAGCTGGACCTTGAATTGGCTCAAAGACACATGCACGCTTGATGCACAGCTAAATGCGGGAATTTACTCAGGCGACTCAAAGGGGAATGACTTTTCGAAAAAAGTGTTTAACCTTTCAATTACATTTTATTTTCTTTCAAAGCTGTCACAGCCCCAGTCAAAAATGCAATACCTCTAGACCTCTGATAAATGTGGTTGTGTGCGCTCTTTTAACCGTATCAAGCTAAATGGGTACTTGTTGAAGAGTATGAACTAGCGGAAGGAACTCTTTAGAGCATATATCTTGACATGACTGCTCGTACAAAAACTGTGATCTCTTAAAACCTCGCAAGGGCGTTGGCACTGCACGCATTTTAAATATAATACAATCACCTCAGGTGCAATCTTAAAGCCAGGATTAAACGTCTGAATTTTGAAGAGTCAACATACAAAATGTTTGCTTAAAAGCACTCTAAATTTAGTCGAAATTCATAATCTAGTTATCCGCGCGCGTAGTGCCCCAACTGGGG 3’<br>Right homology arm (gBlock):<br>5’ CGGAGGTTAAGAAAGCATACGGACGTGCTTGCCTGGCTGTTCATCCTGATAAGGTATGTGACAAGATGTATGCAAAAACTAGGTGGTTTATGTTAATGCAATCTATTTTTAGCATAATGGAACAGAGAATGAAGAGATTGCCAAGCTTATATTTATGGAACTAAACAACGCATGGACAGATTTTGAAAACGATGCCACGCAGCAAAATATGTTTAATGCGTAAAACGCGTCTTATATCTACATATATATATATCACAGTTAGCCCGTATT

	TTCGTACTCTTAGCTGAATCTAATGTAAATTCATGTAAAAGTGTGA GCTTTAATCTTTAAAGTTGAAGTATTGGCATATGTATGCGTATGCA ATTTAGATAGCTCAAACAAACTTTAAAAATTATGAACGCCCGAGG ACATTCTACTTAATTTGCGTAACAGAAAACCCGAATCCAACATTCC CATTGCATCCAACAACAGTCCGCCCAAAAATAACGTCGGAAAGTT AAGAACAAAGCCTTCAGACTTCGGCGACTACCCAATGATTTCACG CCTTATCCATCAAACAATAGAGATCAATTATTCGTGTATATTAATA TACACTTTTTCGCTGTAATTAGATTAATTGCGGTATGCCCTTGACC GATTAGTCAATATATGATTATATGATTAATCTATAAAGTGATGGAA GCAAAAAGTATGGCATTTGTACATAGCTGTTCCGAATATAAAATGT AAAACTAGTAAAAAACCATTTGCTTTTACTTTTTAGCTGTGTACAT AAATAAGCATATTAGAGGTTATCTAAATTCCGTTTCAGCATTTAAT GTTTGCCACTATAAAAATAAATTCTCTATACGTTTCCTTGTGAAAT GCCACTGTTAAAGGTAGAACTGCTCTGTGACTCATTGGTATCCCT GTTTAATACGCTTGCCGGCCACGAAAACTTCTACAATGTTGCGGT CGCTATAACTAGTAACTCGAGT 3’
pBS-KS-attB1-*dAux*^{WT} or *dAux*^{RG}-attB2	N-terminal part of *dAux* is created by gBlock containing overhangs with pBS-KS backbone, the HA-tag and N-terminal part of *dAux* gene:<br>5’ TGCAGGGCCACATCGATAAGAGCCCAGCGTCAAATATCGATAA GAGCACAGCGCCACATATCGATGGTGAGCCAGGCCTGCGCGAT CAAGCTATCGGAGCAAACTGATTCCGCTCCACCCTGCTGAGGAA CCTGCTCCTCCGCAGCCCTGGATTCTTTTTATTTTTTTGTGGCCA CGGCGCTACGAAAATGTTTTAGTTCAGTTAACGGGCGCCAGATAT TTTAGGTGTATCACTTGTCGATTACAGCCTTCTAGCATTGGCCAC CACCAAAATGtacccctacgacgtgcccgactacgccGGCGAGTTCTTTAAGT CGCTCAACCTCAACTACTTCTCCTCTAGCGAGGTCAACGGAGCT GCA 3’<br>Fw and Rv primers to amplify dAux from BAC CH322-22D05:<br>5’ CGAGGTCAACGGAGCTGCAGGAAACGGAGCACCAGAGGCCCT CGGTGGCAGGCTGG 3’ / 5’ ATGATGGTCGACAAGCTTGGATCCC TCGAGGAATTACCTTTAACAGTGGCATTTCACAAGGAAAC 3’<br>Fw and Rv primers for site-directed mutagenesis on pBS-KS-AttB1-dAux-AttB2:<br>5’ GAAAGCATACggtCGCGCCTGCC 3’<br>5’ TTAACCTCGGTCGGTGTCAC 3’

pUAST-AttB1-*dAux*-AttB2	Fw and Rv primer to produce *dAux* with pUAST overhangs: 5' tgggaattcgcggccgcggctcgagATGGGCGAGTTCTTTAAGTCGCTCA 3' / 5' ggttccttcacaaagatcctctagaTTACGCATTAAACATATTTTG CTGC 3'

Supplementary video 1 – 4: Negative geotaxis at 15DO of *dAux*^{WT} , *dAux*^{RG}, *dAux*$^{WT/F956x}$ and *dAux*$^{RG/F956x}$.

Supplementary video 5 – 8: Seizure assay at 5DO of *dAux*^{WT} , *dAux*^{RG}, *dAux*$^{WT/F956x}$ and *dAux*$^{RG/F956x}$.

Supplementary video 9 – 10: Negative geotaxis at 5DO of *dAux*$^{WT/F956x}$ and *dAux*$^{RG/F956x}$ overexpressing Synj in neurons.

Supplementary video 11-12: Seizure assay at 5DO of *dAux*$^{WT/F956x}$ and *dAux*$^{RG/F956x}$ overexpressing Synj in neurons.
