# Supplementary material for: Parkinsonism mutations in DNAJC6 cause lipid defects and neurodegeneration that are rescued by Synj1
Source: NPJ Parkinsons Dis. 2023 Feb 4;9:19. doi: 10.1038/s41531-023-00459-3 (PMC9899244; doi:10.1038/s41531-023-00459-3)
Supplement: Supplementary file 2 — Unprocessed WB scans [file 41531_2023_459_MOESM2_ESM.pdf]

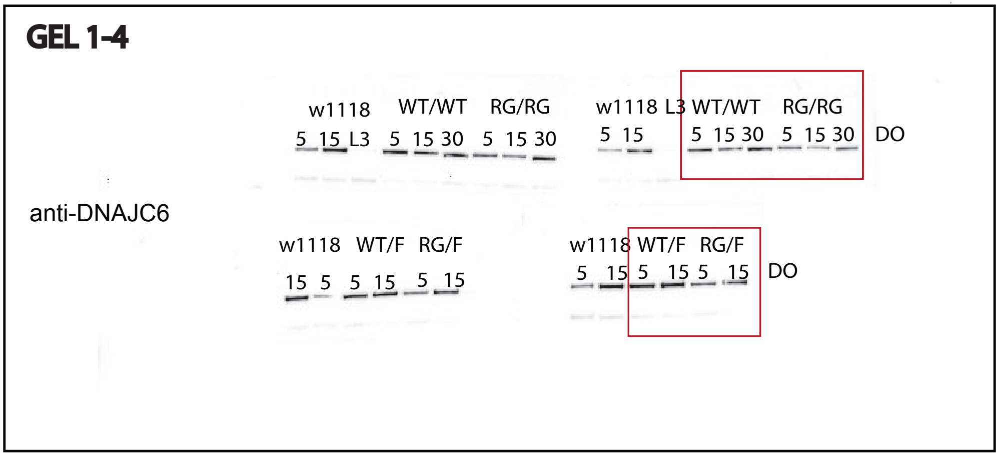

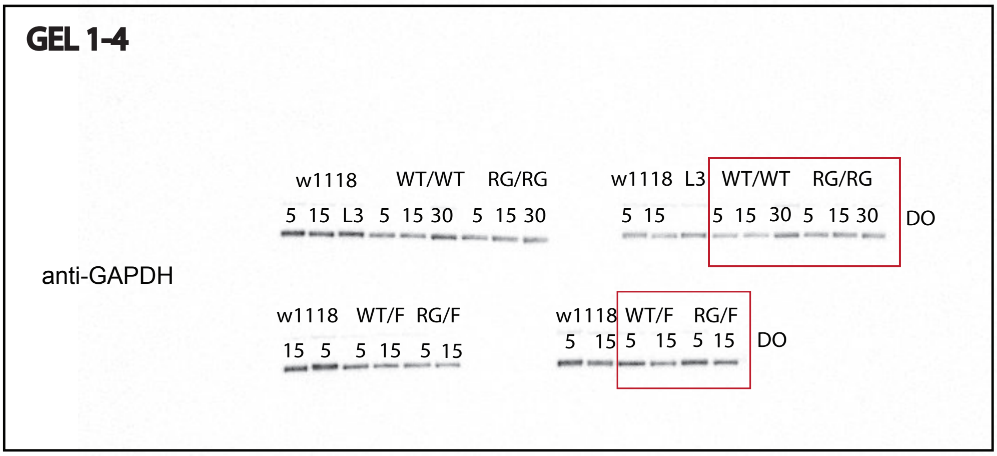

Red boxes indicate what is represented in the paper.

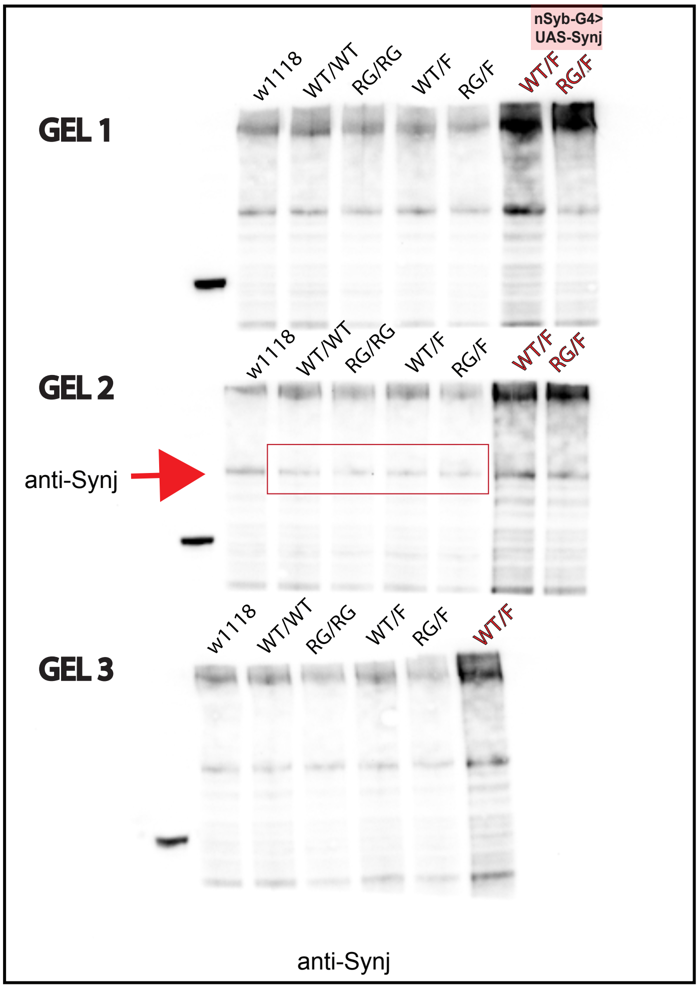

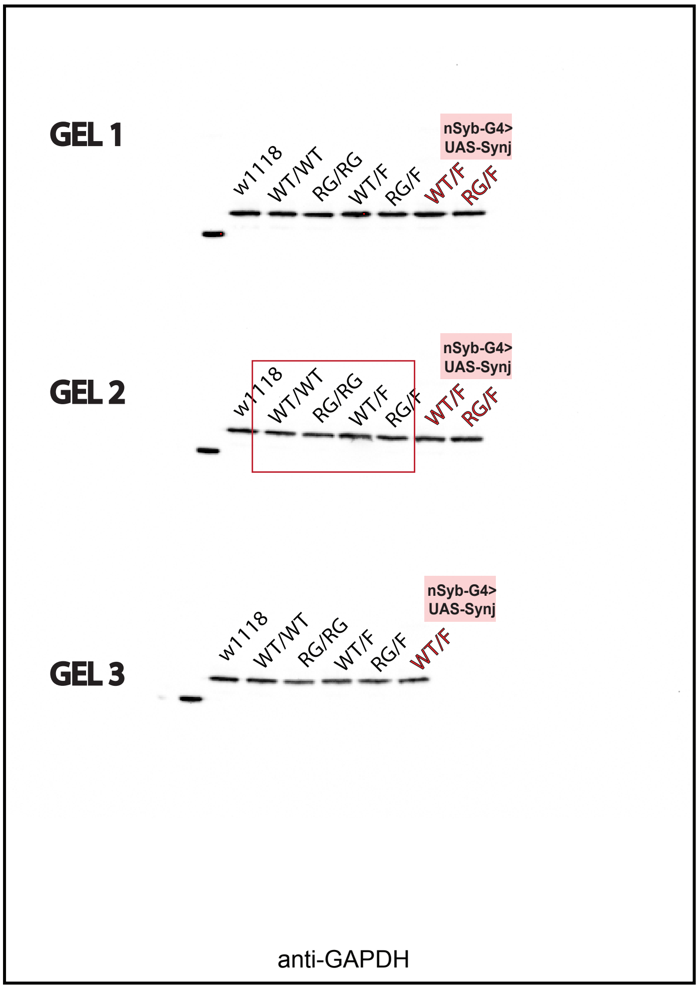

Red boxes indicate what is represented in the paper.
